# Protocol for assessing functional impairments in patients with unilateral and bilateral vestibulopathy: a novel approach to evaluate the impact of vestibular loss in daily life setting

**DOI:** 10.3389/fneur.2025.1704687

**Published:** 2025-12-01

**Authors:** Julie Corre, Gautier Grouvel, Sai Yadnik, Jean-François Cugnot, Sinan Ghavami, Anissa Boutabla, Samuel Cavuscens, Maurizio Ranieri, Raymond van de Berg, Stéphane Armand, Nils Guinand, Angélica Pérez Fornos

**Affiliations:** 1 Division of Otorhinolaryngology Head and Neck Surgery, Geneva University Hospitals and University of Geneva, Geneva, Switzerland; 2 Kinesiology Laboratory, Geneva University Hospitals and University of Geneva, Geneva, Switzerland; 3 Centre of Research on Skeletal Muscle and Movement, Geneva University Hospitals and University of Geneva, Geneva, Switzerland; 4 Division of Clinical Neurosciences, Geneva University Hospitals, Geneva, Switzerland; 5 Division of Otorhinolaryngology Head and Neck Surgery, Lausanne University Hospitals, Lausanne, Switzerland; 6 Division of Vestibular Disorders, Department of Otorhinolaryngology and Head and Neck Surgery, Maastricht University Medical Center, Maastricht, Netherlands

**Keywords:** vestibulopathies, wearable sensors, ecological assessment, daily living tasks, functional assessment, inertial measurement unit (IMU), eye tracking device, plantar pressure insole

## Abstract

**Background:**

Current vestibular assessments typically focus on isolated reflex pathways, failing to reflect the integrative nature of balance control. Consequently, clinical results often do not align with patient-reported symptoms or functional limitations in daily life.

**Objective:**

To develop and present a comprehensive multimodal protocol for assessing functional impairments in patients with unilateral vestibulopathy (UV) and bilateral vestibulopathy (BV) using wearable sensors and ecologically valid daily-life tasks.

**Methods:**

We designed a protocol combining nine inertial measurement units (IMUs), eye-tracking glasses, and plantar pressure insoles to assess participants during 15 standardized tasks reflecting daily activities. Tasks were selected through literature review, validated questionnaires (DHI, VADL), and patient interviews. The protocol is conducted in a semi-naturalistic rehabilitation facility environment to maximize ecological validity while maintaining standardization. We tested feasibility with 60 participants (20 UV, 20 BV, 20 healthy controls).

**Protocol outcomes:**

The protocol successfully demonstrates feasibility across all sensor modalities and task categories. In this paper we describe the methodology used for task selection, the results of task performance in people with unilateral and bilateral vestibulopathy and healthy controls, and the sensor methodology (inertial measurement units, eye-tracking glasses, plantar pressure insoles). Analysis of sensor data will be presented in future papers.

**Conclusion:**

This protocol provides a patient-centered, ecologically valid framework for quantifying vestibular-related functional impairments beyond traditional laboratory settings. The methodology bridges the gap between clinical vestibular testing and lived patient experiences, enabling objective assessment of real-world mobility challenges for personalized rehabilitation and treatment monitoring.

## Introduction

### Background

The vestibular system, located in the inner ear, is a multidimensional motion sensor, mainly responsible for balance, commonly referred to as the “6th sense.” Severe impairments of the vestibular system dramatically affect quality of life (1–5) resulting in a wide variety of significant functional limitations including chronic instability, impaired visual acuity during movement (6), altered environmental/self-perception, or abnormal spatial orientation (7–9). Vestibular deficits make some tasks of daily living challenging or even impossible to perform, triggering imbalance and falls (10–12) especially in older populations (13, 14) and have been associated with cognitive impairments and reduced hippocampal volume due to altered vestibulo-hippocampal integration (5, 6). Moreover, as vestibular function works unconsciously, severe bilateral vestibulopathy (BV) transforms automatically executed tasks into conscious, tiring, efforts. This is worrying considering that functional deficits of the peripheral vestibular system are disabling and prevalent, affecting 15 to 35% of the adult population according to recent estimates (1, 2, 15). The clinical follow-up of vestibulopathies remains a challenge and despite the significant burden imposed by these deficits, current clinical assessments are limited in scope. Indeed, they primarily focus on isolated vestibulo-ocular and vestibulo-spinal reflexes (VOR and VSR, respectively), often measured in laboratory environments that fail to replicate the complex demands of daily activities (10, 14, 15). The vestibulo-spinal system encompasses both the medial vestibulo-spinal pathway (responsible for vestibulo-collic reflexes and head stabilization) and the lateral vestibulo-spinal pathway (responsible for postural stabilization). These tests do not capture the multisensory integration and context-dependent adaptations required for functional balance control (8, 15), and therefore their outcomes correlate poorly with both patient-reported symptoms and perceived handicap (14, 16). As there is unfortunately no curative treatment nowadays for peripheral vestibulopathies, chronic unsteadiness, the most frequent symptom, should be a high priority intervention target to improve quality of life. Emerging wearable technologies—such as inertial measurement units (IMUs), eye-tracking glasses, and foot pressure sensors—offer promising avenues to quantify dynamic stability and gaze behavior in ecologically valid conditions. These tools allow to assess balance and movement patterns during daily-life tasks, providing objective metrics that could serve as relevant ones of functional impairment (16–18). Their use may significantly enhance diagnostic precision, enable remote monitoring, and better inform rehabilitative strategies for patients with vestibular loss.

### Prior work

#### Laboratory-based studies of functional impairments in vestibulopathy

Previous research on functional impairments in vestibulopathies largely focused on specific sensorimotor deficits assessed in controlled laboratory settings. Most studies investigated balance and gait performance using traditional tools such as force platforms, clinical balance tests, or spatiotemporal gait analysis under constrained conditions. Patients with bilateral vestibulopathy (BV) and unilateral vestibulopathy (UV) show marked alterations in postural control and locomotion due to impaired integration of vestibular input with proprioceptive and visual cues (19, 20).

Patients with BV exhibit significant deficits in dynamic stability, particularly under challenging conditions such as eyes closed, concomitant to head movements, or dual tasking. Spatiotemporal gait parameters frequently altered in this population include reduced walking speed, increased stride time variability, increased base of support, and high medio-lateral trunk sway (10, 21, 22). Several dynamic stability biomarkers have been identified in wearable-sensor studies, including increased angular jerk, high root mean square (RMS) of acceleration and angular velocity, and altered harmonic ratios indicating irregular gait patterns (23–25). These parameters reflect the need for compensatory mechanisms in the absence of reliable vestibular input, and correlate with fall risk (26–28).

Similarly, UV patients demonstrate altered postural control and gait asymmetries, especially during head movements or turns (29). While compensation occurs to a greater extent in UV than in BV, residual instability persists in many patients during dynamic tasks, particularly in low-light or visually conflicting environments (30).

In recent studies, our group has contributed to the identification of robust gait and postural metrics using advanced laboratory tools, being among the few to directly compare both BV and UV populations. Corre et al. (19) employed the Central Sensorimotor Integration (CSMI) test to show that BV and UV patients have altered sensory reweighting strategies, with BV patients displaying an increased reliance on proprioceptive input and reduced use of visual cues under dynamic surface conditions. These altered sensory weights distinguished vestibular patients from healthy control subjects with high sensitivity. In parallel, Grouvel et al. (20) and Boutabla et al. (31) demonstrated that both BV and UV patients show reduced gait speed and stride length, with BV patients also exhibiting significantly increased gait variability (GaitSD) and poorer performance on clinical tests such as the Functional Gait Assessment (FGA) and Tandem Walk. Additionally, Grouvel et al. demonstrated altered head stabilization strategies in BV patients, where the head is stabilized relative to the trunk rather than in space, reflecting compensatory adjustments in sensorimotor control.

#### Studies in realistic settings

Despite these laboratory advances, few studies have directly evaluated functional motor behavior during actual real-life tasks. Among these, Mijovic et al. (29) stands out as a pioneering study using wearable sensors to assess head kinematics in a daily-life setting. They demonstrated that vestibular schwannoma patients with documented postoperative UV present a distinct “motion fingerprint” in daily activities, particularly during head movements. However, their analysis was limited to head movements and did not include whole-body motor control, gait parameters or eye movements. Moreover, this study focused exclusively on patients with chronic UV, while it is now well established that patients with BV experience more severe and persistent functional impairments. BV patients typically exhibit greater postural instability (10), higher fall risk (21), poorer gait performance and adaptation (24, 25), and report significantly higher handicap and reduced quality of life compared to UV patients (4, 11). Consequently, studies assessing real-life functional deficits in this more severely affected population are critically needed to better understand their mobility limitations and guide personalized rehabilitation strategies.

While earlier studies such as Wuehr et al. (24) and Schniepp et al. (21) laid important groundwork by using mobile systems to quantify gait variability and coordination in BV patients, these approaches now serve primarily as methodological references. These foundational findings have since inspired more technologically integrated and ecologically focused assessment protocols (25, 27, 32).

#### Sensor technologies tested and gaps in current approaches

The use of wearable inertial measurement units (IMUs) and pressure insole sensors in ecological settings has gained increasing attention, as it allows for the identification of objective functional biomarkers directly translatable to clinical practice and rehabilitation monitoring. For example, Jabri et al. (33) explored multi-sensor gait analysis in patients with vestibular hypofunction. However, their study was limited to a six meters walkway and did not include tasks simulating activities of daily living in a semi-naturalistic environment. Furthermore, these findings, while important, are mostly derived from highly controlled laboratory settings and may not fully represent real-world mobility demands. They do not account for environmental unpredictability, attentional shifts, or multitasking inherent to daily life. Thus, despite the availability of objective laboratory biomarkers of dysfunction, the ecological validity of these findings remains limited.

A notable gap in current assessment approaches is the absence of comprehensive eye movement analysis during functional tasks. Eye movements, often overlooked in functional assessments, are critical for maintaining visual stability and orientation, and may serve as sensitive markers of impairment when assessed alongside postural and locomotor behavior in daily-life scenarios (34–36). To our knowledge, no previous study has comprehensively integrated wearable inertial sensors, eye-tracking, and plantar pressure analysis for the functional assessment of UV and BV patients under ecological conditions.

#### Scientific gaps addressed and clinical relevance

Based on these results, it appears highly relevant to design an assessment protocol that goes beyond conventional gait analysis by capturing whole-body movements in more naturalistic conditions, evaluating the whole spectrum of vestibulopathy (uni- and bi-lateral). Given the central role of head stabilization in vestibular compensation—as well as the interplay between head and eye movements for gaze control and navigation—particular attention should be paid to the dynamics of head motion and oculomotor behavior during functional tasks.

Moreover, in recent interventions such as vestibular implants (11, 37) and noisy galvanic stimulation (38–41), quantifiable gait and balance metrics appear important to quantify the benefit of the treatment. Despite these advances, to our knowledge, there remains a lack of comprehensive multimodal studies assessing the full-body motor patterns of vestibular patients during tasks mimicking daily life, including transitions, obstacle negotiation, dual tasks and gaze tasks. Our study addresses this gap by combining wearable IMUs, eye-tracking, and foot pressure insoles in a structured battery of real-life tasks.

#### Task-set selection

To establish a challenging yet ecologically valid and achievable set of tasks reflecting the daily-life difficulties encountered by patients with vestibular loss, we developed our task set through an iterative, multi-source selection process combining existing literature, validated clinical tools, and direct patient input. We initially referred to the work of Mijovic et al. (29) which represents, to our knowledge, the only prior study using wearable sensors to assess vestibular patients in a daily-life setting. They demonstrated that head motion patterns during daily-life activities differ significantly in patients with chronic UV compared to healthy control subjects and proposed that such motion “fingerprints” can be used for functional assessment. However, their study focused exclusively on chronic UV patients (not BV), assessed only head kinematics (not whole-body movements, gait, or eye movements), and included 10 tasks that served as our initial foundation but required substantial adaptation and expansion for our broader objectives. Additionally, items were extracted from the validated French versions of the Vestibular Disorders Activities of Daily Living Scale (VADL) (42) and the Dizziness Handicap Inventory (DHI) (43), both of which are widely used to quantify the impact of vestibulopathies on autonomy and perceived handicap. These instruments provided a patient-reported framework of activities that individuals with vestibular loss find challenging in daily life.

To complement this theoretical framework and ensure that the selected tasks were meaningful from a patient-centered perspective, we conducted semi-structured interviews with three patients suffering from severe BV. These patients were selected based on the severity of their symptoms and their long-standing clinical follow-up, which allowed for rich experiential feedback. Critically, these interviews revealed that several daily activities they found most challenging were not included in Mijovic’s original task set, leading to the addition of tasks specifically relevant to BV patients (e.g., walking in the dark—requiring heavier reliance on vestibular and proprioceptive input single; negotiating a stepladder—requiring foot placement precision on narrow steps; carrying a tray with water—requiring smooth upper body control; walking on a narrow wood beam—i.e., with a reduced base of support). Conversely, some tasks from Mijovic et al. or initially considered by our team were deemed less relevant, too easy, or logistically problematic, and were subsequently excluded (see Supplementary Table 2 for complete list of discarded tasks and rationale). Their contributions helped us identify which daily activities were perceived as most challenging and limiting. This approach ensured that our task selection not only aligned with existing assessment scales but also addressed daily life limitations reported by BV and UV patients themselves, as recommended in participatory design methodologies in rehabilitation science (23).

The final set consisted of 15 tasks, classified into three levels of difficulty (easy, intermediate, difficult). The activities were designed to cover a broad spectrum of balance demands, including transitions, gait, dual-tasking, and obstacle negotiation, thereby increasing sensitivity to subtle impairments in motor control and gaze stabilization. The final 15-task set therefore represents a synthesis of: (1) literature foundation from Mijovic et al. adapted to our broader objectives, (2) items from validated clinical instruments (DHI, VADL), (3) direct patient input identifying gaps and confirming relevance particularly for BV patients, and (4) iterative refinement through multiple rounds of review and pilot testing. The task set was not defined from the outset; instead, it resulted from an iterative selection process during which several versions were tested, reviewed, and refined to ensure relevance, feasibility, and complementarity. Tasks were evaluated based on their ecological validity, their ability to challenge vestibular-related postural control, and their compatibility with the constraints of the hospital setting. The task list is presented in Table 2. Tasks initially considered but ultimately excluded due to ethical, logistical, or participant-related reasons are documented in Supplementary Table 2 along with the rationale for each exclusion decision, providing transparency regarding the iterative refinement process.

**Table 2:** Description of the 15 tasks included in the protocol.

Task		Description	Materials	Challenge
1-Bed		The participant lays flat on the bed; then sits up and finally stands up. 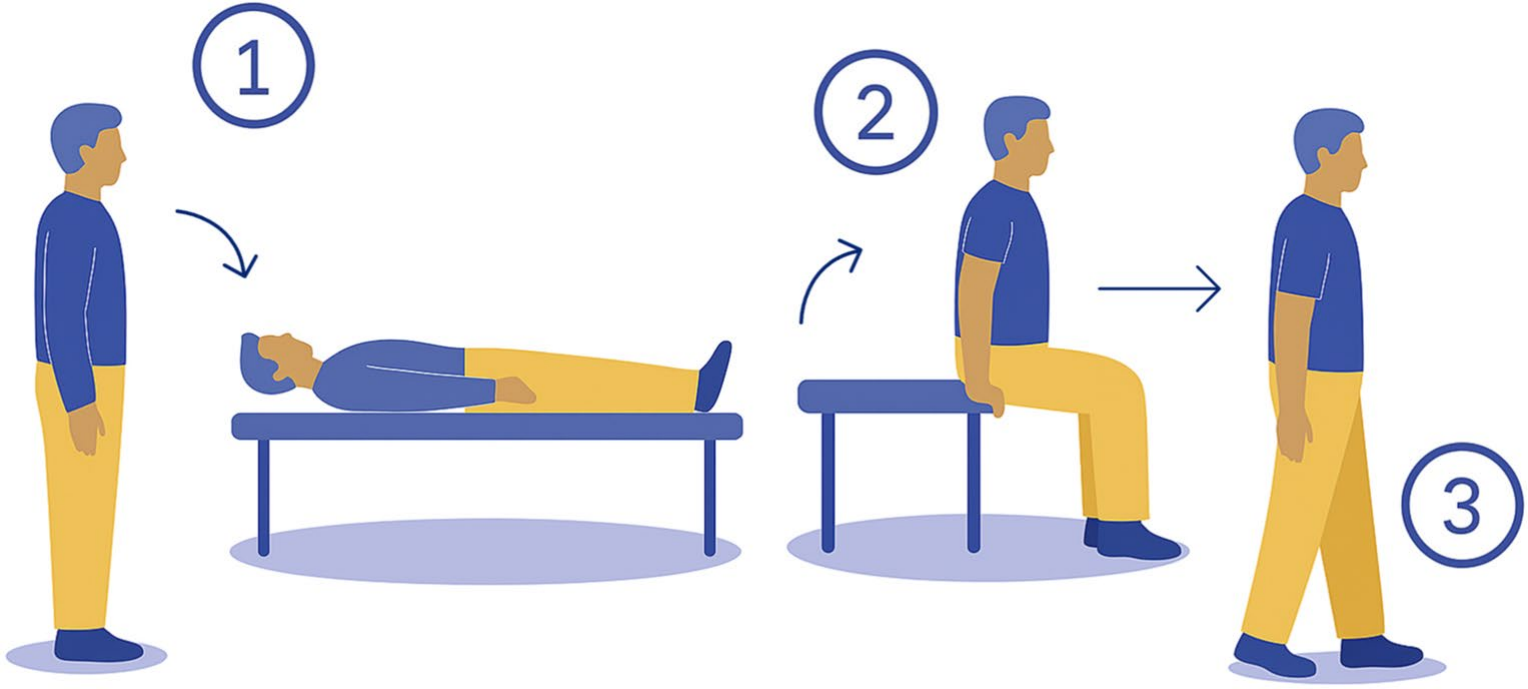	Flat examination table	Whole body movements Dynamic balance
2-Pants		Standing up and without external support, the participant puts a pair of loose pants one leg at a time and then takes it off. 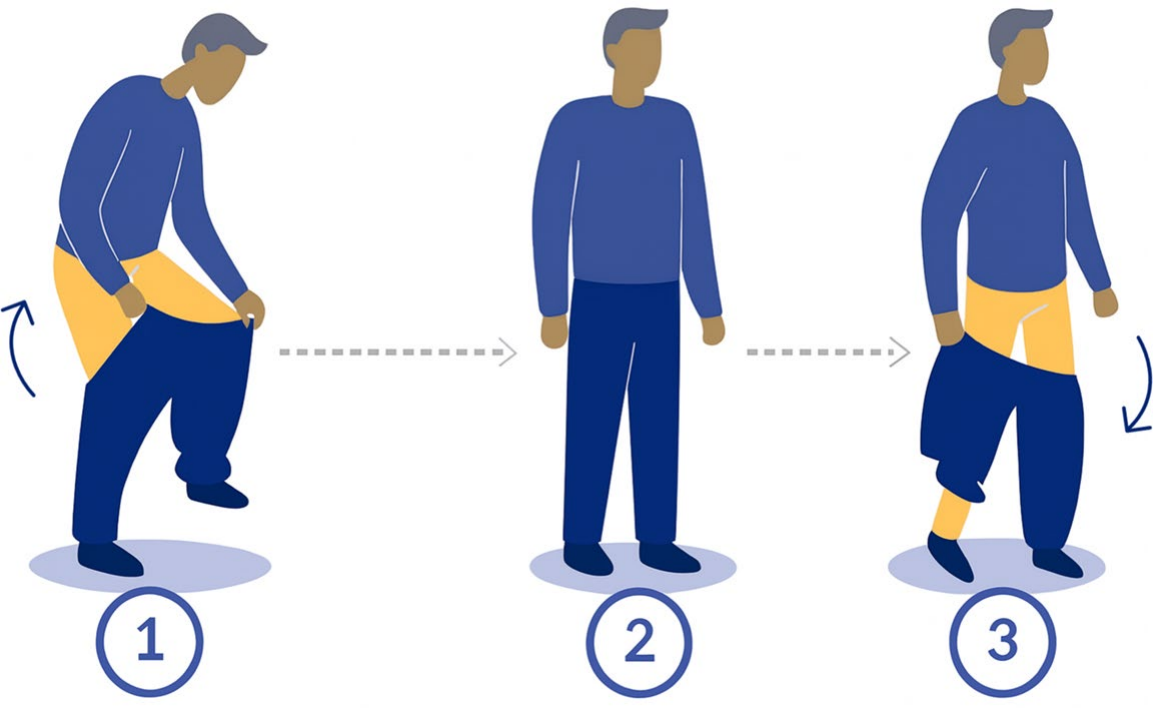	XL scrubs	Reduction of base of support Shifting of CoM
3-Shoes		The participant takes off and puts back on a pair of sports shoes without sitting or kneeling. 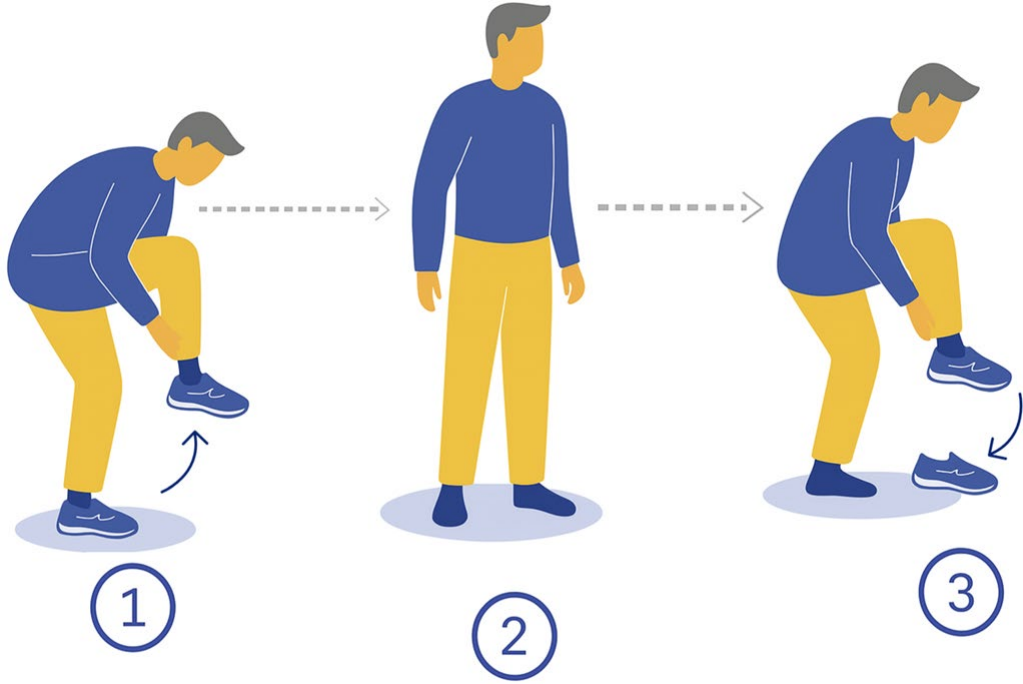	Standardized sport shoes at the subject size	Reduction of base of support Head motion
4-Sorting		The participant sorts plastic dishes from a storage box into a shelving unit according to color and size. 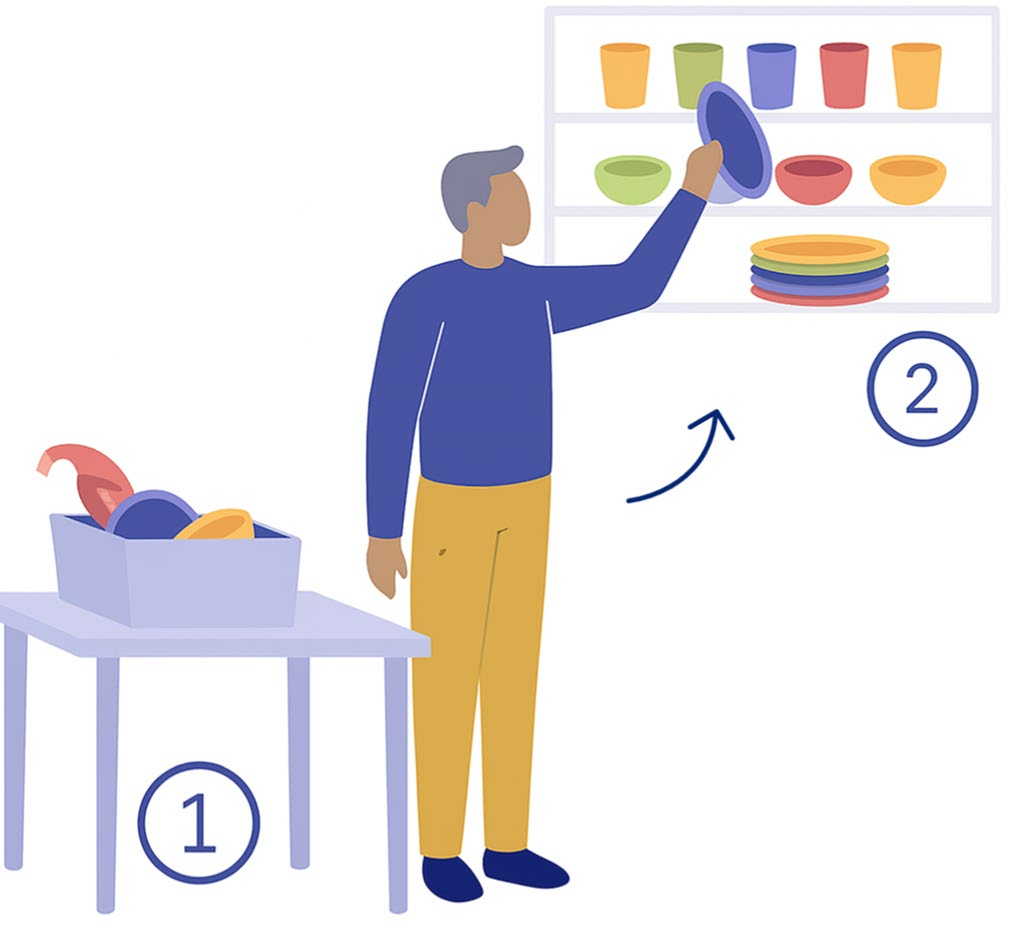	4 × 4 shelving unit 3 plastic cups, bowl and plates of 5 different colors (total 45)	Rapid head movement
5-Heavy load		The participant carries a fully loaded bucket (5 kg) on one hand while walking on a straight line, turns around, changes the bucket to the other hand and comes back to the departure point (20 m round trip). 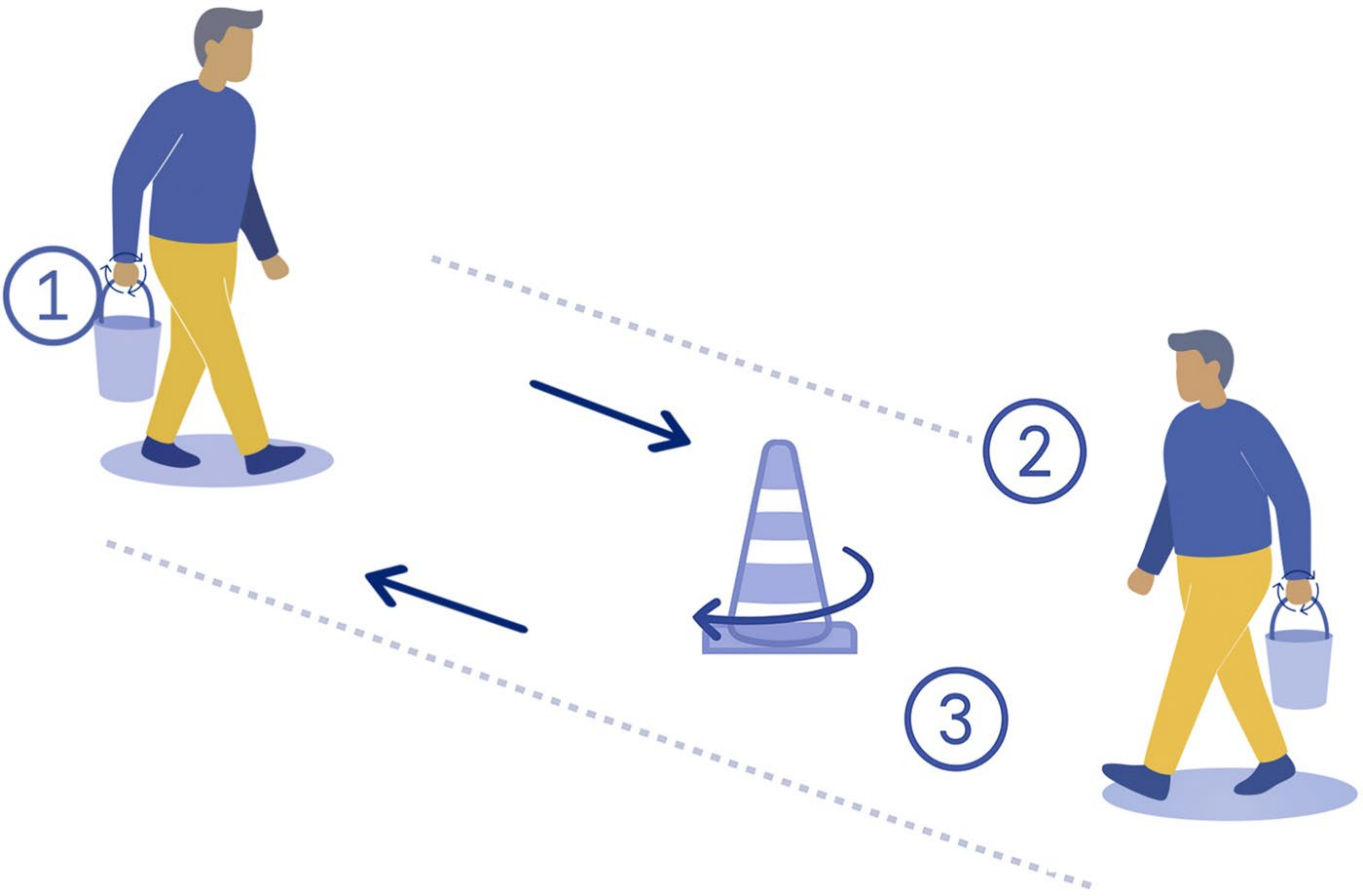	Loaded bucket (5 kg)	Shifting CoM
6-Bus		The participant calls a mock bus, gets in, sits down, calls a stop, and gets off. 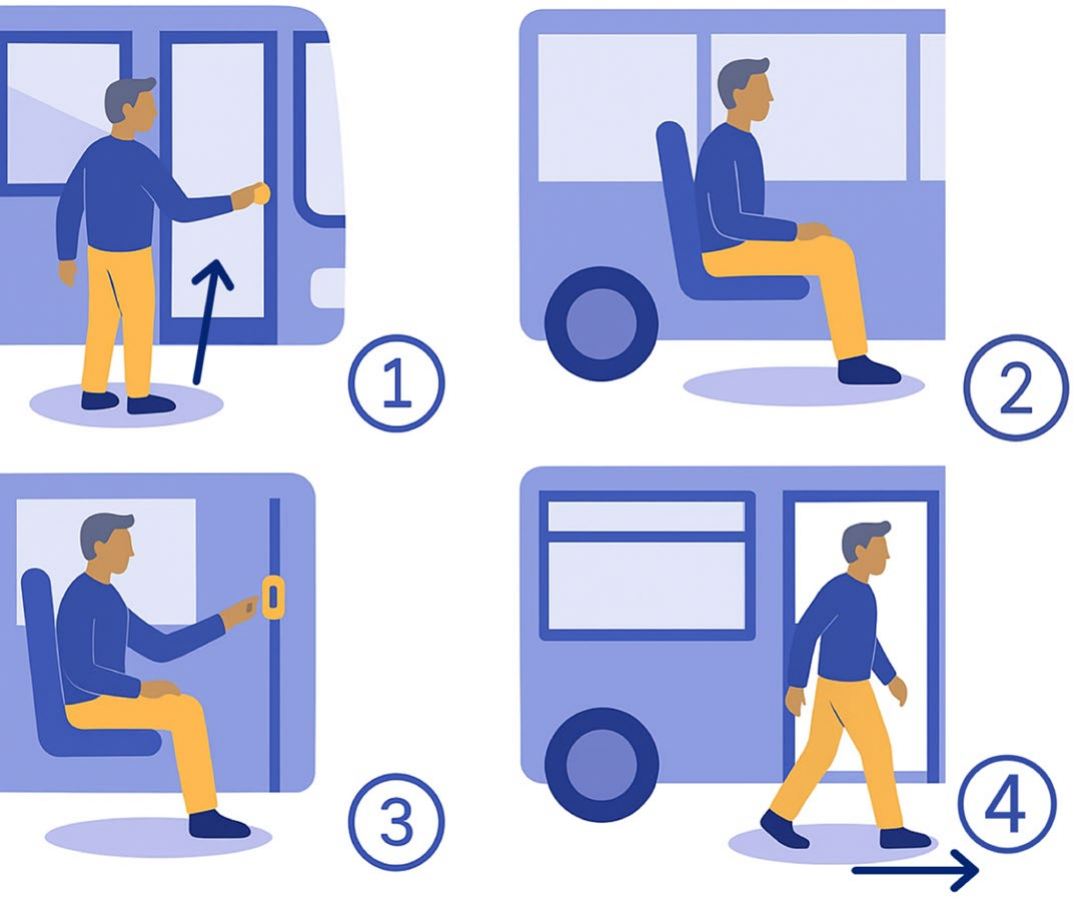	Mock bus	Whole body movements Dynamic balance
7-Stairs up	Down	The participant goes up 8 consecutive straight stairs and then down 6 consecutive spiral stairs without holding to the handrail (if possible). 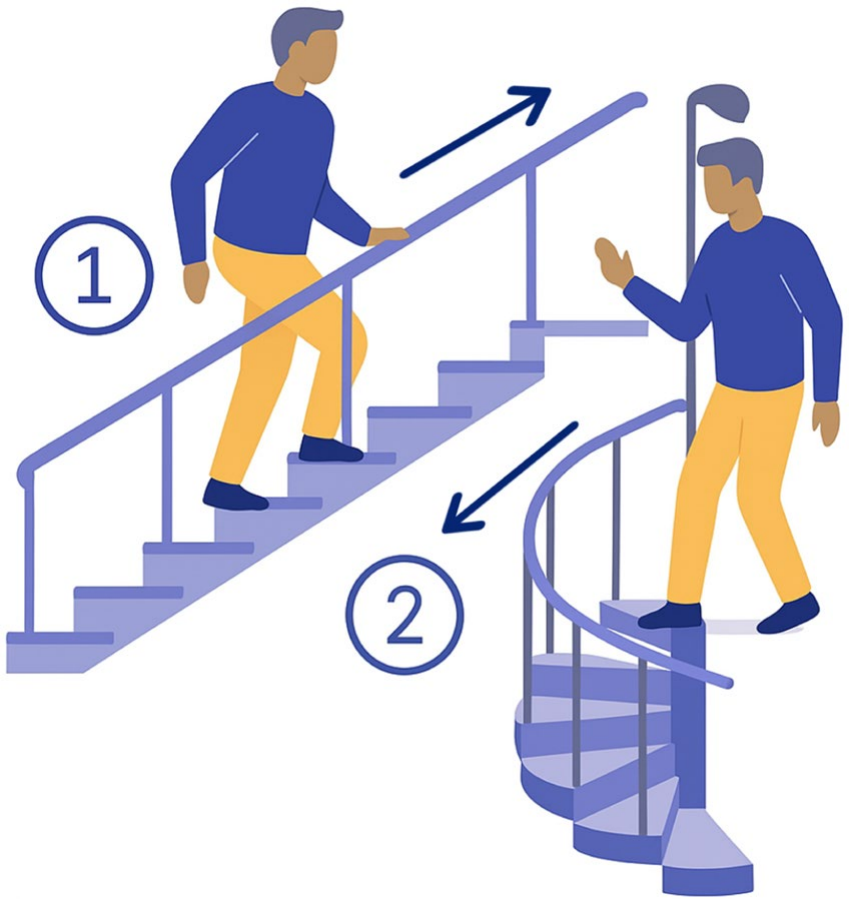	7 consecutive stairs	Shifting CoM
8-Uneven grounds		The participant walks on a paved, rugged road (15 m). 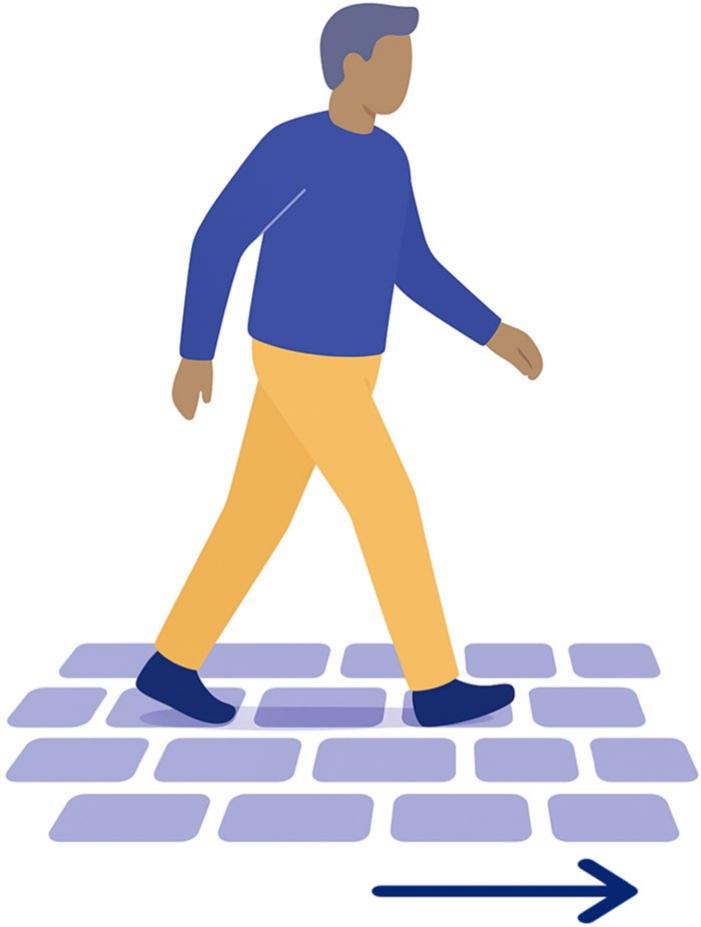	15 m of an uneven ground, preferably cobbled road	Walking on unstable ground (modification of proprioceptive inputs)
9- Stepladder		The participant climbs 5 steps of a ladder then walks back down. 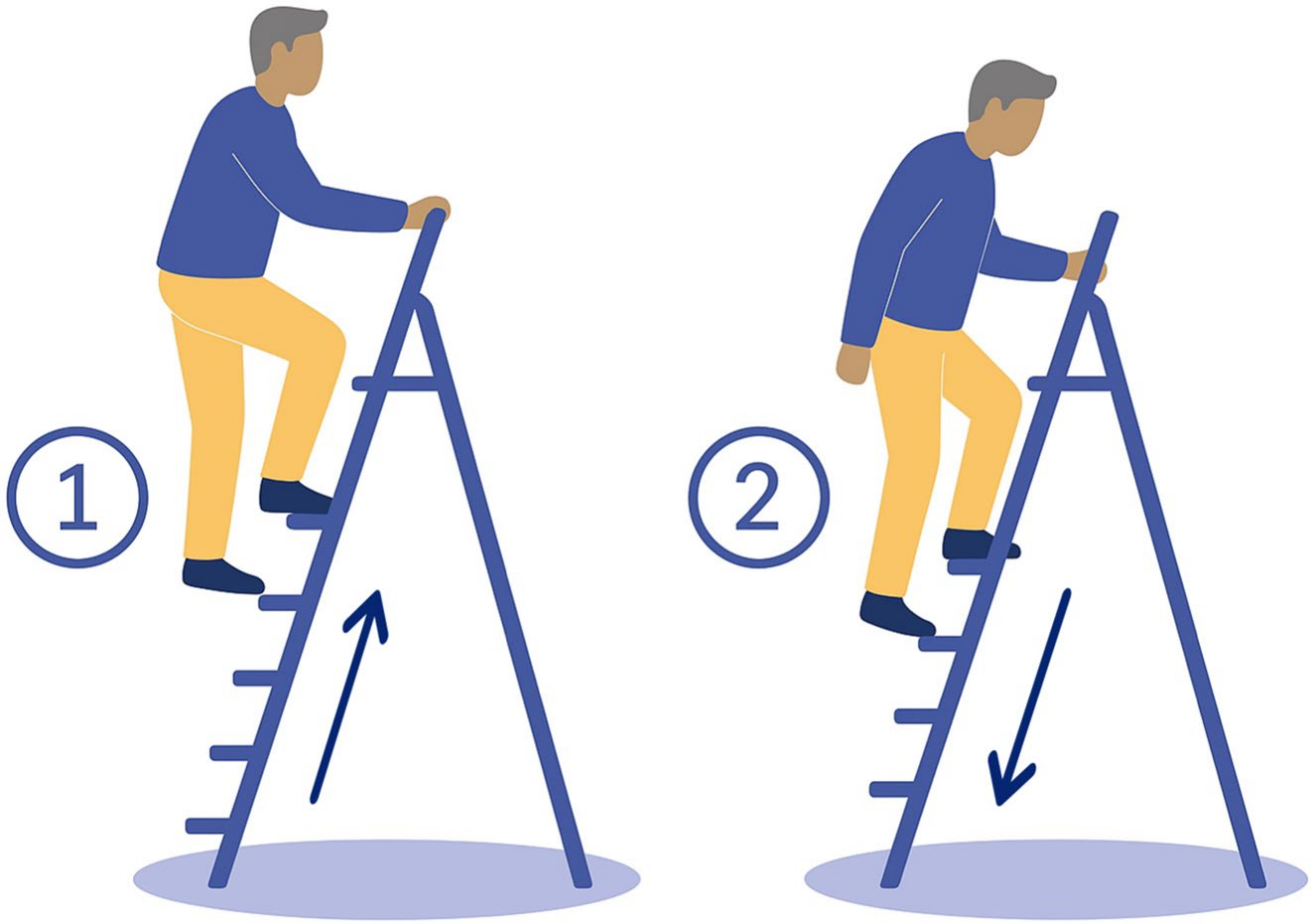	5 steps ladder, with 8 cm width steps, 21.3 cm spaced from each other	Modification of feet proprioception Dynamic balance
10-Tray		The participant carries a glass of water on a tray over 12 m without dropping it. 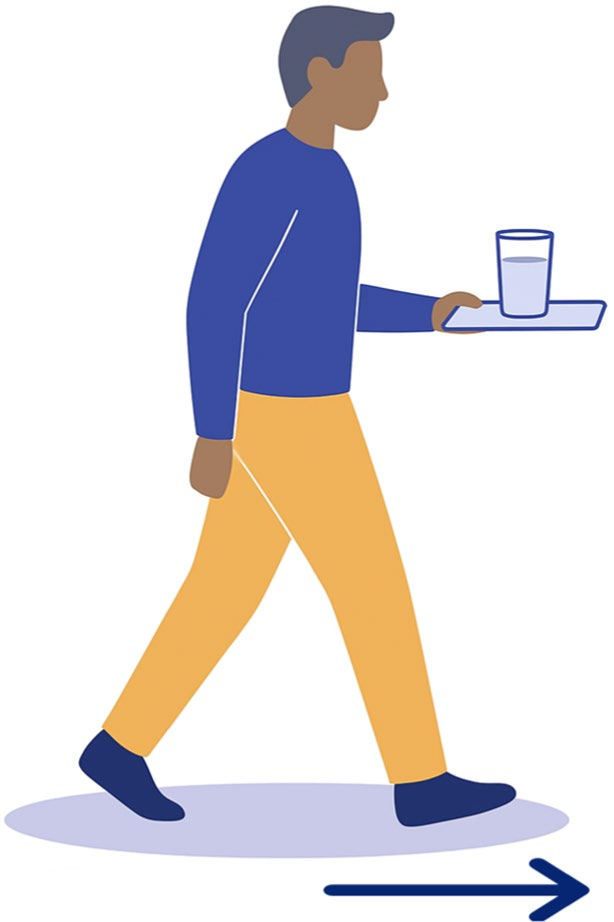	1 standard cafeteria tray and a glass of water (250 mL)	Dynamic balance
11-Walk		The participant walks on a straight 12 m line at their most comfortable pace. 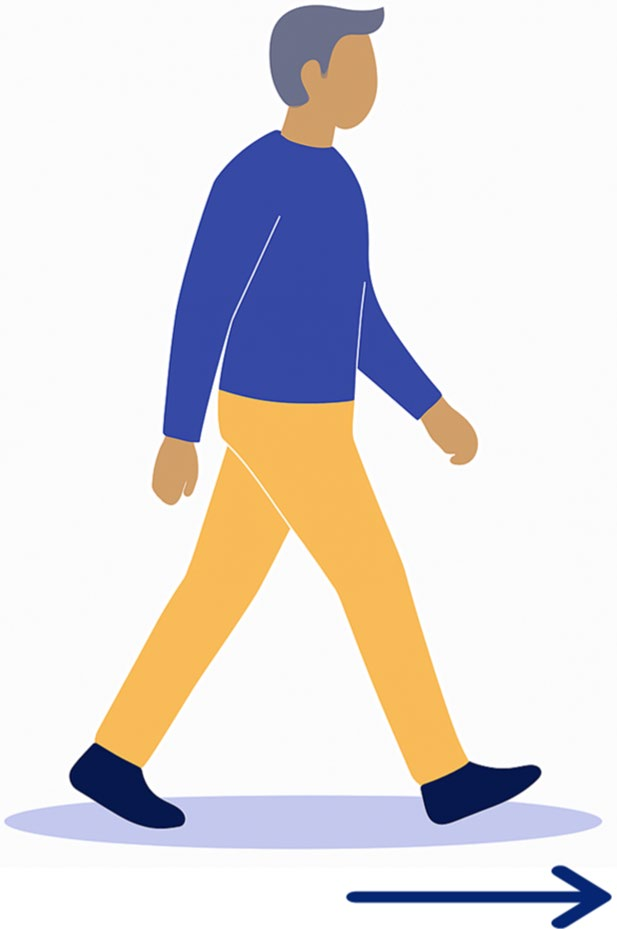		
12-Wood Beam		The participant walks on a 5 m narrow wood beam (50 cm wide), turns around and comes back. 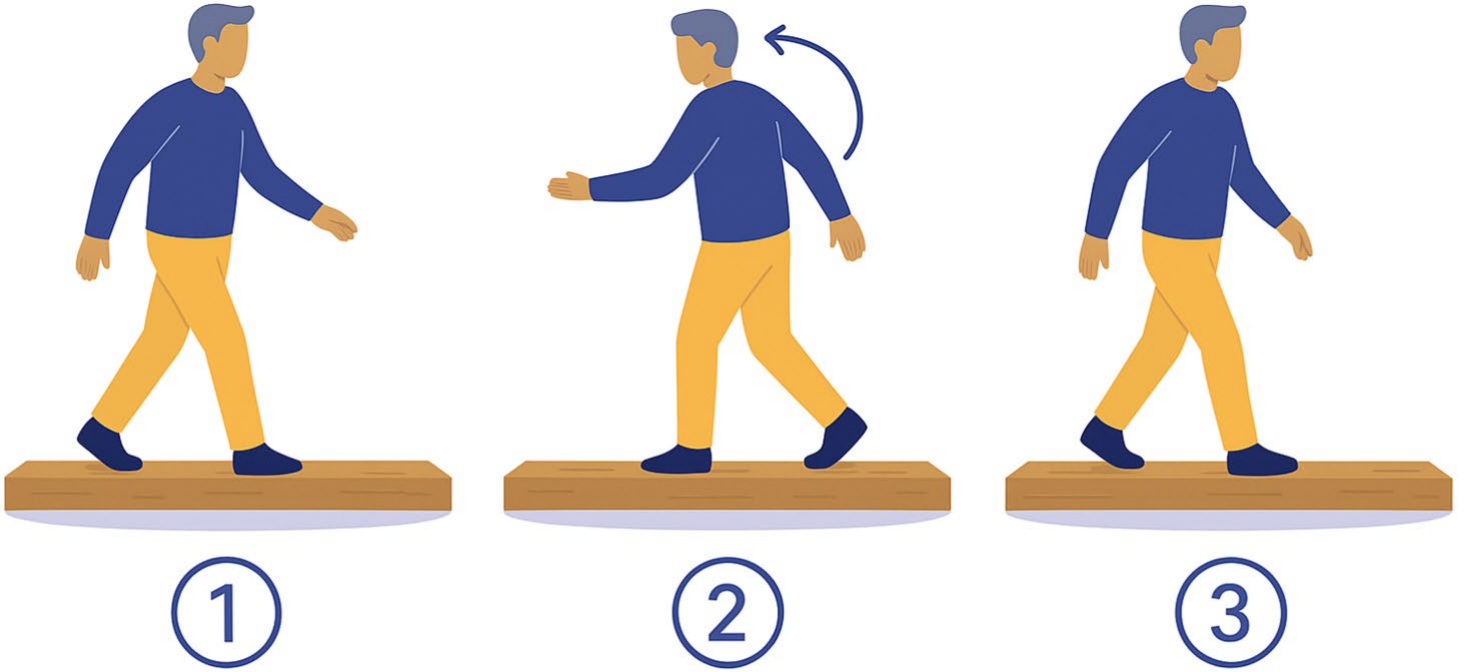	Wood beam, 5 m long and 50 cm width	Reduction of base of support Dynamic balance
13-Uphill	Down	Participant walks over & down a 15^0^ elevation ramp with eyes open/closed without holding to the ramp 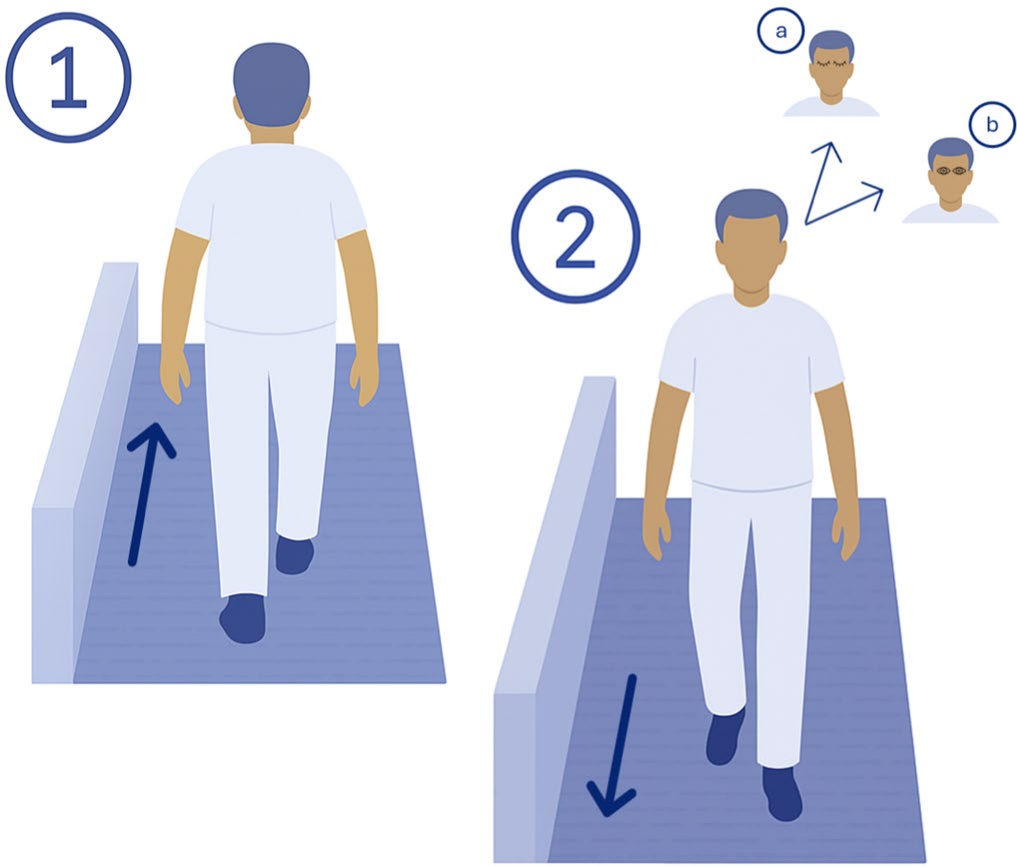	Ground elevation (2 sets of 15 degrees elevation over 2 meters)	Modification of feet proprioception Absence of visual inputs
14-Picture recognition		Three photographs depicting everyday life scenes are mounted on the window surfaces of the experimental room (1 m separation between them). The participant is instructed to walk along a straight 10-meter pathway, turning at the end to return to the starting point (total distance per trial: 20 meters). While walking, the participant observes the displayed images. Upon completing the walking task, the participant is asked to verbally describe the pictures in as much detail as possible. 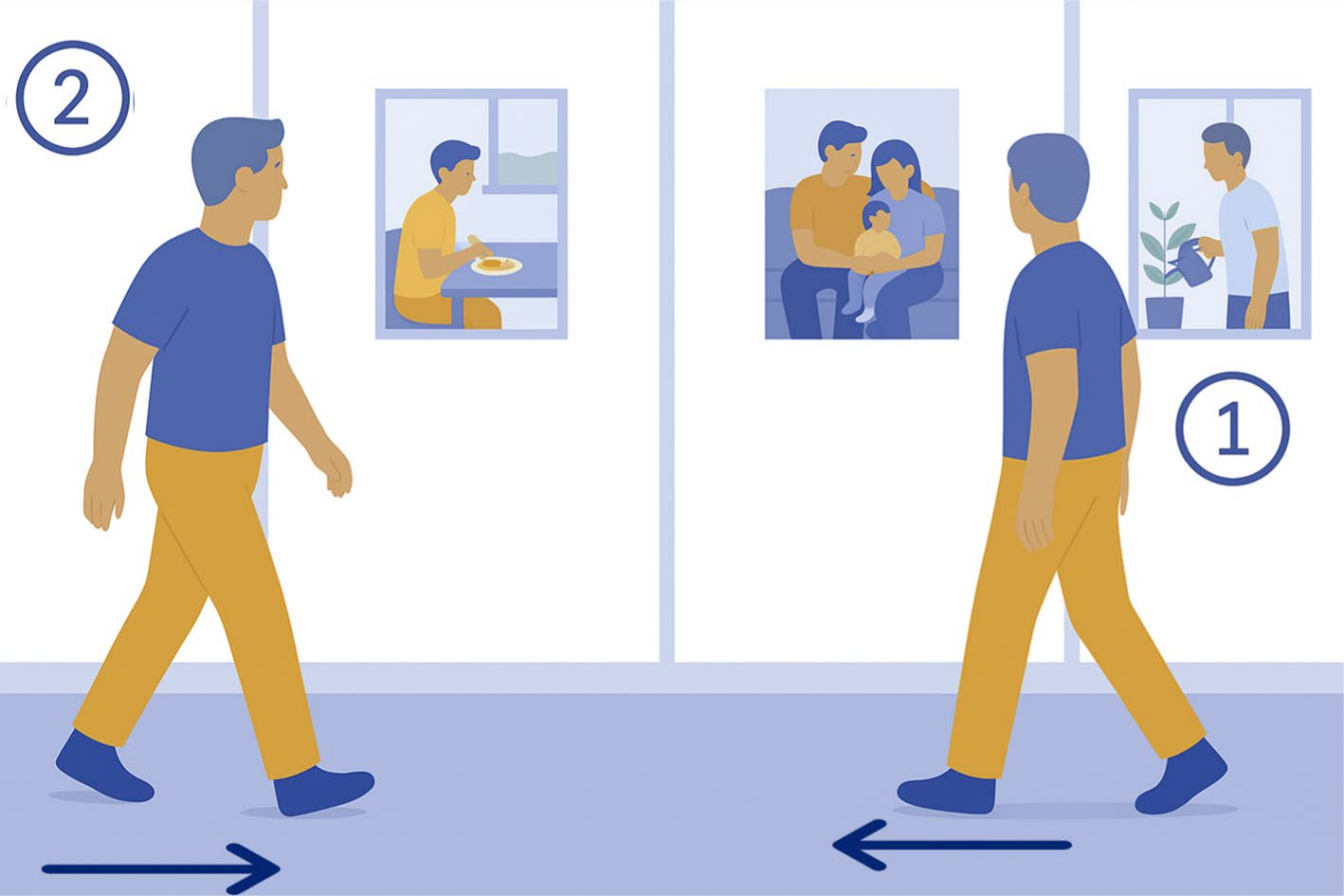	Large set (for randomization) of A4 printed and plasticized pictures	Dual Task
15-Walk in the dark		The participant walks on a straight 12 m line while wearing dark glasses. 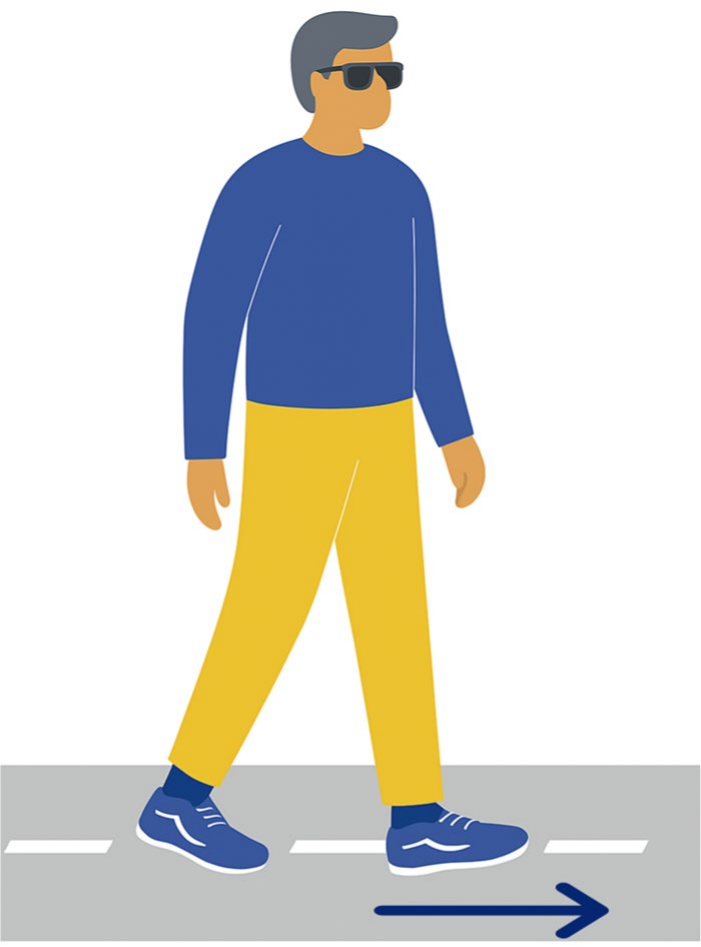	Athermal welding protection goggles DIN 5	Absence of visual inputs

For each task, its description with an illustration, the materials required for setup, and the specific postural or sensorimotor challenges targeted are detailed. The task set was designed to replicate a broad spectrum of daily-life activities that engage different aspects of balance control, including dynamic stability, proprioceptive adaptation, visual dependency, reduction of base of support, and dual-tasking. The difficulty level of each task was estimated based on patient interviews and pilot testing: tasks perceived as easy are highlighted in green, intermediate in yellow, and difficult in red. XL, extra-large size; CoM, Center of Mass.

#### Choice of the environment

To maximize ecological validity while ensuring logistical feasibility and safety, we initially considered multiple locations. We ultimately selected the rehabilitation facility of Beau-Séjour Hospital (Geneva University Hospitals, Geneva, Switzerland) for several key reasons. This facility—commonly referred to as a rehabilitation track—is a specialized assessment and training environment designed for functional rehabilitation of patients before discharge home. It is typically used by physical therapists and occupational therapists to rehabilitate patients who have experienced strokes, amputations, traumatic injuries, or prolonged hospitalization resulting in reduced mobility. The facility is specifically equipped with real-world environmental features and challenges that patients will encounter in daily life: residential stairs (both straight and spiral configurations), ramps of varying inclines, uneven surfaces including cobblestones, household furniture and appliances (kitchen counters, beds, chairs), doorways, narrow passages, and other obstacles simulating home and community environments. This rehabilitation facility was particularly well-suited for our study for several reasons. First, it authentically simulates the diversity of postural challenges encountered in daily life rather than providing the artificial, uniform surfaces typical of laboratory settings. The variety of terrains and supports closely replicates real-world conditions—stairs, ramps, uneven ground, household items—allowing assessment of functional capacity in ecologically valid contexts. Second, its central location in Geneva facilitated easier access for both participants and the research team, and was more practical logistically, particularly given the amount of equipment that had to be transported and set up. Finally, the chosen track provided a large and versatile space, allowing participants to perform a wide variety of tasks independently, which reduced social pressure and stress during testing (access was limited to participants and the research team). This type of hybrid setup (semi-standardized but in a daily life setting) aligns with recent calls for “ecological assessment” frameworks in vestibular research, which emphasize the importance of contextualizing sensorimotor function beyond the laboratory (32).

#### Pilot tests with patients and control subjects

Finally, to ensure both the feasibility of the protocol, regarding time needed to complete the set, its difficulty, and the pertinence of the chosen tasks, 3 BV patients and 3 Healthy Subjects (HS) executed the complete protocol before launching the study.

#### Aim

This protocol combines the use of wearable sensors, including IMUs, eye-tracking glasses, and foot pressure insoles, to capture the dynamic balance and gaze behavior of BV and UV patients compared to HS. All sessions were video recorded. Fifteen daily-life tasks were used to challenge postural stability and assess gaze patterns. The specific aims of this manuscript are to:

1. Demonstrate the feasibility of applying multimodal wearable sensors (IMUs, eye-tracking, pressure insoles) during ecologically valid tasks in patients with vestibular loss, documenting technical challenges and data quality achieved.
2. Present task completion patterns across groups, documenting which tasks could be performed by all participants versus those representing functional limitations particularly in bilateral vestibulopathy patients.
3. Characterize demographic and clinical profiles (DHI, SF-36) of the study population to establish baseline vestibular-related handicap and quality of life.
4. Provide comprehensive sensor methodology to enable reproducibility and inform future analytical papers examining sensor-derived biomarkers of functional impairment.

Ultimately, this feasibility study establishes that multimodal wearable sensor assessment during daily-life tasks is implementable in patients with vestibular loss, identifies which tasks are achievable versus prohibitively challenging, and provides the methodological foundation for subsequent analyses of movement, gaze, and pressure patterns.

## Methods

### Study design

This prospective study was conducted in accordance with the principles of the Declaration of Helsinki and was approved by the Cantonal Commission for Research Ethics of Geneva (BASEC-ID: 2024–02394). Written informed consent was obtained from all participants and data are being processed confidentially in compliance with applicable ethical standards.

### Participants

The study population consisted of 44- to 83-year-old adults, divided into 3 groups, namely patients suffering from either uni or bilateral vestibulopathy (UV and BV groups, respectively) and healthy subjects (HS, control group). The assessments were performed at the Beau-Séjour rehabilitation facility. Patients were recruited from the outpatient consultations of the otoneurology unit of the Geneva University Hospitals (HUG) and Lausanne University Hospital (CHUV). The BV group encompassed individuals suffering from severe BV, diagnosed in agreement with the consensus criteria of the Barany Society (31). Briefly, diagnostic criteria for BV included unsteadiness and/or oscillopsia during walking or head movements, and a reduced bithermal caloric response (sum of bithermal maximal peak slow-phase velocity < 6°/s bilaterally) and/or bilaterally reduced video head impulse test (vHIT) gains < 0.6. The UV group included individuals presenting a unilateral deficit for at least 6 months, documented by vHIT gain values below 0.6 for the lateral semicircular canal of the affected ear, and vHIT gain values above 0.8 in the other ear. UV patients were recruited at least 6 months after acute symptoms. Finally, all HS presented normal vHIT gain values for all semicircular canals (vHIT gain values above 0.8). The exclusion criteria for all groups were the presence of psychiatric disorders, stigmata of vascular dementia, blindness, obesity, or recent (less than a year) hip prosthesis or knee replacement. All study participants were over 18 years of age. All participants included in our study provided written informed consent. Demographic variables (age, sex, height, body mass index) and etiology were documented for all participants. Clinical characterization included standardized questionnaires assessing vestibular-related handicap (Dizziness Handicap Inventory, DHI) and health-related quality of life (SF-36 Health Survey) as described in the Questionnaires section below. Between-group comparisons of demographic variables were performed to confirm adequate matching of the three groups. Complete demographic and clinical characteristics are presented in Table 1. At the beginning of each session, participants were systematically asked about their current physical state, including whether they were experiencing any pain, discomfort, or other factors that might affect their ability to perform the tasks safely. This informal screening allowed identification of acute issues, though we acknowledge that chronic musculoskeletal conditions such as arthritic joint pain were not systematically assessed using standardized pain questionnaires or documented through comprehensive medical history review.

**Table 1:** Main demographic and anthropomorphic characteristics of the sixty patients included in this study.

	Healthy subjects (HS)	Unilateral vestibulopathy (UV)	Bilateral vestibulopathy (BV)	Statistics (groups comparison) *p* values*
Participant number (N)	20	20	20	–
Sex (N)				
Female	10	10	12	–
Male	10	10	8	–
Age: years, mean (SD)	57.9 (5.3)	59.5 (5.5)	60.7 (11.5)	0.557
Range	45–65	46–66	44–83	
Height: cm, mean (SD)	172.1 (8.4)	173.4 (10)	168.3 (8.6)	0.192
BMI: kg/m^2^, mean (SD)	24.3 (4.1)	24.7 (3.4)	25.3 (3.5)	0.711
Etiology (N)				
Idiopathic		5	14	
Meniere’s disease		2	1	
Schwannoma		9	1	
Traumatic		2	0	
Ototoxic		0	3	
Zona		1	0	
Cyst		1	0	
Cogan		0	1	
DHI	N/A	28 (17)	48 (19)	< 0.001
SF-36				
MCS	48.5 (15.4)	40.1 (15.4)	43.2 (11.2)	0,134
PCS	52.7 (9.6)	50.1 (9.2)	39.8 (12.2)	HS-BV < 0.001; UV-BV 0.037; HS-UV: 0.537

Data are presented as mean (standard deviation) for continuous variables and counts for categorical variables. Statistical comparisons: Between-group differences for continuous variables (age, height, BMI) were assessed using one-way ANOVA. DHI scores were compared between UV and BV groups using independent samples *t*-test. SF-36 component scores (MCS, PCS) were compared across the three groups using Kruskal-Wallis test due to non-normal distribution, with post-hoc pairwise comparisons performed using Dunn’s method when overall significance was detected. A *p*-value <0.05 was considered statistically significant. For PCS, pairwise comparisons revealed significant differences between HS-BV (*p* < 0.001) and UV-BV (*p* = 0.037), but not between HS-UV (*p* = 0.537). Abbreviations: SD, standard deviation; BMI, body mass index; DHI, Dizziness Handicap Inventory; SF-36, Short Form-36 Health Survey; MCS, Mental Component Score; PCS, Physical Component Score; N/A, not applicable.

### Sample size

Using data from a recently published study by our group (34), we calculated the sample size required to detect significant differences between the three groups (power 0.95, *α* 0.05). In this previous laboratory study comparing the same three populations (BV, UV, and HS), we identified walking speed during comfortable gait as a robust discriminating parameter between groups. The observed values were: BV: 1.09 ± 0.22 m/s, UV: 1.05 ± 0.17 m/s, HS: 1.29 ± 0.09 m/s. Sample size calculation was performed using G*Power (35) software with a one-way ANOVA to compare the three groups. Based on these walking speed differences; the analysis yielded an effect size *f* = 0.31 and indicated a required sample size of 16 participants per group to achieve 95% power. For additional statistical robustness, we aimed for 20 participants per group.

### Data collection

#### Task description

The 15 tasks of the set were performed in the order displayed in Table 2. Each task was designed to reflect a specific aspect of functional mobility commonly challenged in individuals with vestibular loss. The table outlines the name of each task, the description of the task, the materials required for setup, and the specific balance-related challenge it was intended to elicit (e.g., dynamic stability, dual-tasking, proprioceptive demand, reduced visual input). The specific instruction provided to the participant is presented in Supplementary Table 1.

#### Procedure

**Figure 1:**
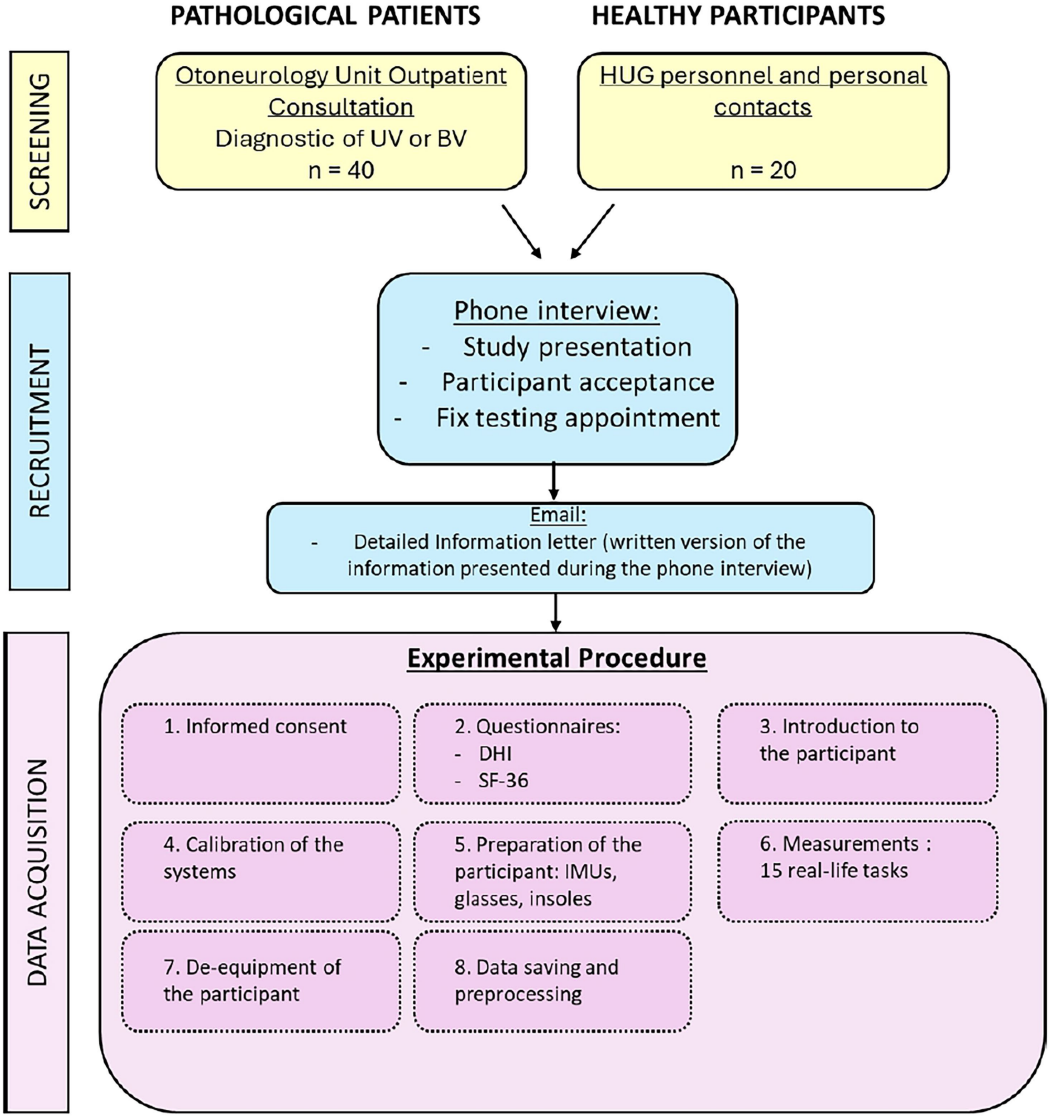
Flow diagram of the study. UV, Unilateral Vestibulopathy patient; BV, Bilateral Vestibulopathy patient; HUG, Geneva University Hospital; DHI, Dizziness Handicap Inventory; SF-36, 36-Item Short Form Health Survey and IMU, Inertial Measurement Unit.

The data were collected over one single session of approximately 1 h duration per participant. All sessions were supervised by two operators: one who equipped the participant and then ensured data recordings; the other who provided instructions to the participant and ensured safety. The flow diagram of the study is described in Figure 1.

The following procedure was systematically adopted:

1) *Introduction to the participant:* Participants were welcomed and briefed on the session objectives, the nature of the tasks to be performed, and the approximate duration. Consent and safety procedures were reviewed again, and participants were encouraged to ask questions. Particular emphasis was placed on informing them of the possibility of pausing, skipping a task or even stop the experiment at any point if discomfort arose.
2) *Calibration of the systems:* The calibration of the IMUs was performed by the manufacturer, and no calibration was required before the measurement. The calibration of the pressure insoles was performed in accordance with the manufacturer’s instructions, including both zeroing and calibration procedures. The zeroing process involved an automatic mode that continuously monitors and compensates for sensor offsets and drifts caused by factors such as shoe-lacing and temperature changes. Additionally, a manual zeroing function was available through the software (OpenGO, Moticon GmbH). The calibration routine, conducted via the app, included very slow walking and static postures (OpenGO, Moticon GmbH). Eye tracking calibration was made once the participant was fitted with the glasses, during which the participant held a calibration target at arm’s length and stared at it (Tobii Pro Lab Software, Tobii AB, Danderyd, Sweden).
3) *Preparation of the participant:* Each participant was equipped with a full set of wearable sensors, including 9 IMUs placed on the head, torso, sacrum, left and right wrists, left and right thighs, and left and right feet; eye-tracking glasses; and two plantar pressure insoles inserted into the standardized shoes among participants. The devices were secured using adjustable straps or belts, and fit was double-checked to ensure that they were comfortable for the participant to wear and that the sensors provided clear and reliable data (avoiding sensor movements on clothes). If the participant required prescription glasses that were incompatible with the eye-tracking glasses, the latter were not used for the session. In such cases, only IMU and plantar pressure data were collected. This exception was noted in the session record to inform data interpretation.
4) *Measurements:* Participants performed the 15-task protocol under supervision, with verbal instructions and demonstrations provided before each task if needed. Task performance was also video recorded. After each task, participants could rest as needed to avoid fatigue or dizziness.

*Fatigue management:* To minimize fatigue effects, several measures were implemented. Total protocol duration was limited to approximately 1 h, and participants were explicitly informed they could rest between tasks as needed. Task order was standardized (Table 2), generally alternating from simpler to more complex activities to distribute physical and cognitive demands. Operators actively monitored participants for signs of fatigue throughout the session. Notably, no participant requested a formal break during the protocol, suggesting the duration and task progression were well-tolerated.

### Task completion and data classification

An important methodological distinction was made between tasks not performed due to functional limitations and technical missing data. This distinction is clinically meaningful and fundamental to the ecological validity of our protocol.

*Tasks not performed due to functional limitation*: When participants declined a task due to symptom severity or perceived inability to perform it safely, this was recorded as “task not performed” (either “Did Not Start” – DNS when the participant did not start the task or “Did Not Finish” – DNF when the participant started the task but interrupted it before completion) rather than missing data. This represents a clinically meaningful functional limitation and constitutes an important outcome measure. For example, several BV patients declined Task 2-Pants (putting on pants while standing) because they felt unable to maintain balance on one leg, and others declined Task 12-Wood Beam due to fear of falling. All decisions to skip or decline tasks were entirely patient initiated, based on their own assessment of safety and symptom severity. The research team never prevented or discouraged task performance for safety reasons, though participants were always explicitly informed of their right to decline any task at any point without explanation or consequence.

Tasks most frequently declined by participants (see Table 3) included:

**Table 3:** Task completion rates across groups.

Task		Number of participants who completed the task		
		HS	UV	BV
1-Bed		20	20	20
2-Pants		20	20	16 (4 DNS)
3-Shoes		20	20	18 (2 DNS)
4-Sorting		20	20	20
5-Heavy load		20	20	20
6-Bus		20	20	20
7-Stairs up & down		20	20	20
8-Uneven grounds		20	20	20
9- Stepladder		20	20	19 (1 DNS)
10-Tray		20	20	20
11-Walk		20	20	20
12-Wood Beam		20	20	15 (3 DNS; 2 DNF)
13-Uphill	Down	20	20	20
14-Picture recognition		20	20	20
15-Walk in the dark		20	20	20

Task completion represents the number of participants who attempted and completed each task. Numbers less than 20 indicate either patient-initiated task declination (DNS) due to perceived inability or safety concerns related to vestibular symptoms or patient-initiated task termination before completion (DNF) because the task proved too difficult for the capabilities of the participant. All declination decisions were made by participants; no tasks were prevented by the research team. Task non-completion reflects clinically meaningful functional limitations rather than missing data. DNS, Did not Start the task. DFN, Did Not Finish the Task.

- Task 2-Pants: 4/20 BV patients declined (unable to balance on one leg)
- Task 3-Shoes: 2/20 BV patients declined (difficulty bending and balancing)
- Task 9-Stepladder: 1/20 BV patients declined (fear of falls on narrow steps)
- Task 12-Wood Beam: 5/20 BV patients declined (fear of falls, reduced base of support)

Notably, no HS or UV participants declined any tasks.

*Technical missing data*: In contrast to functional task non-completion, technical data loss occurred due to equipment or protocol-specific factors unrelated to participants’ functional abilities. These technical issues are comprehensively documented in Table 4, which presents detailed information on:

**Table 4:** Technical data availability by sensor modality.

Sensor modality	HS (*n* = 20)	UV (*n* = 20)	BV (*n* = 20)	Reason for data unavailability
IMUs	20	20	19	BV: 1 participant - calibration failure affecting all tasks
Eye-tracking: session-level issues	19	16	19	HS: 1 participant - prescription glasses required UV: 2 participants - technical equipment failure; 2 participants - prescription glasses required BV: 1 participant - prescription glasses required
Eye-tracking: Task 14-Picture Recognition additional losses	11/19†	12/16†	16/19†	All groups: Additional participants required prescription glasses specifically for this task demanding high visual acuity (8 HS, 4 UV, 3 BV)
Eye-tracking: protocol design	N/A	N/A	N/A	Task 15-Walk in dark used obscuring goggles (all participants) Task 13-Uphill/Down included eyes-closed phase
Pressure insoles	20	20	20	No technical issues

*†Denominator = participants with functioning eye-tracking equipment for other tasks.* This table documents technical data loss unrelated to participants’ functional capacity. Numbers represent participants for whom sensor data were technically obtainable. *IMUs*: 1 BV participant experienced persistent calibration failure despite multiple attempts, resulting in complete loss of IMU data across all tasks. *Eye-tracking—session-level issues*: Some participants could not use eye-tracking for any tasks due to: (1) technical equipment failure (2 UV participants), or (2) requirement for prescription glasses incompatible with the eye-tracking device (1 HS, 2 UV, 1 BV). These participants performed all tasks but without eye-tracking data. *Eye-tracking—Task 14 specific*: Task 14-Picture Recognition required participants to identify and describe detailed images displayed at distance, demanding clear visual acuity. Among participants with otherwise functioning eye-tracking equipment, an additional 8 HS, 4 UV, and 3 BV participants elected to use their prescription glasses specifically for this task, temporarily precluding eye-tracking. These participants had eye-tracking data for all other tasks. *Eye-tracking—protocol design*: Eye-tracking was impossible by design for Task 15-Walk in the dark (obscuring goggles worn by all participants) and during the eyes-closed descent phase of Task 13-Uphill/Down. *Pressure insoles*: All pressure insole units functioned properly throughout all testing sessions with no technical failures. *Critical distinction*: This table documents only technical limitations. Participants who declined tasks due to symptom severity (functional limitation) are documented in Table 3, not here, even if this resulted in no eye-tracking or IMUs data being collected for that task.

- IMU calibration failure (1 BV participant, affecting all tasks)
- Eye-tracking unavailability due to session-level equipment issues, Task 14-specific prescription glasses requirements, and protocol design constraints
- Pressure insole data quality (no technical issues)

Complete details including exact numbers per group, specific reasons for data unavailability, and task-by-task breakdown are presented in Table 4 and described in the Results section.

This distinction is clinically important because inability to perform a task reflects the real-world impact of vestibular loss on functional capacity, providing valuable information about activity limitations and participation restrictions as defined by the International Classification of Functioning, Disability and Health (ICF) framework. These task non-completion data will be analyzed as outcome measures in subsequent studies examining the relationship between specific functional limitations and disease severity, quality of life measures, and clinical vestibular test results.

### Materials and parameters

#### IMUs

Nine wireless IMUs (Physilog6S, MindMaze, Lausanne, Switzerland) sampled at 128 Hz were placed on the participants’ head, chest, sacrum, wrists, tights, and feet and secured with appropriate sport belts. They recorded 3-Dimensional (3D) linear accelerations with a range of ± 16 g, and 3D angular velocities with a range of ± 2,000°/s. The IMUs were switched on and off using a mobile app, which also controlled the start and stop of each recording to ensure that only the task itself was captured, excluding any intermediate periods. The Physilog6S system has been validated for gait analysis in clinical populations with excellent test–retest reliability (ICC > 0.90 for spatiotemporal parameters) and strong concurrent validity against laboratory-based motion capture systems (e.g., Vicon Motion Capture System, Vicon Motion Systems Ltd., Oxford, UK) (34, 37).

#### Eye-tracking

The eye-tracking glasses (Tobii Pro Glasses 3, Tobii AB; Stockholm, Sweden) were used to track eye movements and gaze behavior. This wearable device included four eye cameras (2 per eye) and multiple infrared illuminators (8 per eye) integrated into the eyeglass frame to continuously record eye position and pupil data (position and size). A high-definition scene camera (1,920 × 1,080 resolution, 25 fps) located in the center of the glasses captures the participant’s field of view. The system also included an IMU comprising an accelerometer (sampled at 100 Hz), a gyroscope (sampled at 100 Hz), and a magnetometer (sampled at 10 Hz) to record head movement in 3D. Eye movement data were combined with head position to compute gaze vectors, which were then mapped onto the video footage from the scene camera. The eye-tracking data were sampled at 100 Hz. The Tobii Pro Glasses 3 system has demonstrated accuracy of <1° under optimal conditions and <3° during dynamic head movements (40), with reliability validated in ambulatory settings.

#### Foot pressure insoles

Two pressure insoles (OpenGo Sensor Insoles 3, Moticon ReGo AG, Munich, Germany) sampled at 100 Hz were used to measure feet plantar pressures. These insoles were composed of 16 pressure sensors, a 3D accelerometer and a 3D gyroscope. The total area covered by pressure sensors ranged from 7.6 mm^2^ to 16.5 mm^2^ depending on sensor insole sizes (adapted to participant’s shoe size). The insoles were switched on just before being placed in the shoes, and switched off at the end of the measurement, thus recording the entire session. Plantar pressure insole systems, including Moticon sensors, have shown good validity against force platforms (r = 0.81–0.99) and test–retest reliability (ICC 0.69–0.97) in gait analysis (44–47). While validation specifically in vestibular populations is limited, these sensors provide objective measurement of plantar pressure distribution and temporal gait parameters relevant for functional mobility assessment.

#### Video recordings

Subjects were recorded while performing the tasks using a camera (GoPro HERO9 Black, GoPro, San Mateo, USA).

*Video Analysis Methodology*: Video recordings were analyzed using a standardized scoring grid for observable instability cues developed through systematic literature review of validated balance and gait assessment tools. The scoring grid was adapted from established clinical instruments including the Functional Gait Assessment (48), Berg Balance Scale (49), Performance-Oriented Mobility Assessment (50), and published observational criteria for vestibular gait deviations (20–22). The grid documents observable instability events including stops, trips and slips, leans, compensatory movements, and other signs of imbalance during task performance.

All video recordings were independently scored by two trained raters: (1) the primary investigator (JC) who conducted the data collection, and (2) an independent expert rater (from RvdB’s team) specialized in the phenomenology of vestibular disorders who was blinded to participant group assignment and clinical characteristics. Both raters completed scoring independently without consultation. Inter-rater reliability between the two independent scorings will be assessed using intraclass correlation coefficients (ICC) for continuous variables and Cohen’s kappa for categorical variables. The complete scoring grid, inter-rater reliability results, and analysis of instability patterns across groups and tasks will be presented in a dedicated future paper (number 4) focusing on behavioral adaptations and compensatory strategies.

Time required to complete the task was also documented.

#### Questionnaires

Finally, before the start of the experimental session, all participants completed standardized self-report questionnaires. These tools were chosen to capture subjective aspects of vestibular loss and overall health-related quality of life, allowing for comparison with objective performance metrics.

For patients with UV or BV, two questionnaires were administered:

1) Dizziness Handicap Inventory (DHI): The DHI is a validated 25-item questionnaire specifically designed to assess the self-perceived handicap caused by vestibular loss. It includes three subscales:

- *Functional* (9 items): assesses the impact of dizziness on daily activities (e.g., walking, reading, social participation).
- *Emotional* (9 items): captures psychological consequences such as frustration, fear, or depression.
- *Physical* (7 items): reflects symptom triggers linked to motion, position changes, or head movements.

Responses are scored as “Yes” (4 points), “Sometimes” (2 points), or “No” (0 points), for a total score ranging from 0 to 100. Higher scores indicate a greater perceived handicap with the following severity classifications: No handicap: 0–15 points; Mild handicap: 16–34; Moderate handicap: 35–54 points and severe handicap: ≥ 55 points.

This instrument was selected because it reflects the patient’s functional and emotional adaptation to vestibular symptoms, which may not always align with objective deficits, especially in chronic cases.

1) *SF-36 Health Survey (Short Form-36):* The SF-36 is a generic, widely used instrument to assess health-related quality of life across eight domains: Physical Functioning, Role Physical, Bodily Pain, General Health, Vitality, Social Functioning, Role Emotional, and Mental Health. Each domain is scored on a 0–100 scale, with higher scores indicating better self-perceived health. The SF-36 provides both a physical and mental component summary score, which aggregate the eight domains using a standardized scoring algorithm (66). We calculated PCS and MCS using Swiss normative reference data (67) to ensure culturally appropriate comparisons. Domain scores were standardized against Swiss population norms, weighted by factor-derived coefficients, summed, and transformed into norm-based T-scores (mean = 50, SD = 10), where scores below 50 indicate below-average health status. Its inclusion allowed for a broader contextualization of the patient’s experience and facilitates comparisons with HS.

For healthy control participants, only the SF-36 was administered to avoid unnecessary burden and to focus on general well-being measures in this reference group.

By combining the DHI and SF-36 in the pathological groups, we aimed to capture both condition-specific and general health perceptions. These subjective data will serve as a basis for correlation analyses with the sensor-derived parameters, to evaluate the ecological and clinical relevance of our multimodal assessment protocol. They will also provide insight into the disparity that often exists between clinical test results and patient-reported disability.

#### Additional clinical and lifestyle information

For patients with UV or BV, additional structured questions were administered to capture rehabilitation history and lifestyle context:

- Vestibular rehabilitation engagement: Prior and current vestibular physiotherapy participation, duration, frequency, and patient-reported satisfaction with therapy outcomes.
- Physical activity history: Type, frequency, and intensity of physical activity and sports participation before vestibular symptom onset, and whether these activities were maintained, modified, or discontinued post-onset.
- Home-based rehabilitation: Adherence to home exercise programs, barriers to continuation, and perceived effectiveness
- Functional impact: Supplementary questions on how vestibular symptoms specifically impact daily activities, social participation, and overall life satisfaction.

These data provide important context for interpreting compensatory strategies and will be analyzed in relation to observed functional performance and sensor-derived parameters.

### Data analysis

*Demographic and clinical comparisons:* Between-group differences in continuous demographic variables (age, height, BMI) were assessed using one-way ANOVA. DHI scores were compared between UV and BV groups using independent samples t-test (HS group was not assessed with DHI). SF-36 component scores (PCS, MCS) were compared across the three groups using Kruskal-Wallis test. When overall significance was detected (*p* < 0.05), post-hoc pairwise comparisons were performed using Dunn’s method with adjustment for multiple comparisons. Statistical analyses were performed using SigmaPlot version 16 (Systat Software, Inc., San Jose, CA, USA) with significance threshold set at *α* = 0.05.

### Intended data analysis

This protocol paper describes methodology for multimodal data collection. Sensor data analyses will be presented in dedicated future papers. Here we briefly outline the overall analytical strategy and publication plan.

### Analytical approach overview

The analytical strategy progresses from single-modality characterization to multimodal integration:

*Single-modality analyses*: Initial analyses will establish robust parameters from each sensor modality independently to validate data quality and identify discriminative parameters. For IMUs: kinematic smoothness, movement intensity, spatiotemporal gait metrics. For eye-tracking: saccade/fixation characteristics, gaze path metrics, head-gaze coordination. For pressure insoles: center of pressure dynamics, weight distribution, temporal gait parameters. Video analysis will characterize compensatory behaviors through structured observational scoring.

*Multimodal integration*: Multimodal integration analyses will examine how parameters from different sensor modalities jointly characterize functional impairments.

*Clinical validation and protocol refinement*: All sensor-derived parameters will be correlated with standard clinical vestibular tests, patient-reported outcomes (DHI, SF-36), and disease characteristics to evaluate ecological and clinical validity. We will also determine the minimal combination of sensors, sensor locations, and tasks needed for effective clinical assessment, informing the development of a streamlined protocol optimized for routine clinical implementation.

*Covariate considerations*: Age will be included as a covariate in all primary analyses to account for potential age-related effects on motor performance and sensory integration that may be independent of vestibular function.

*Publication strategy and rationale*: Sensor data analyses will be disseminated through a series of papers examining: (1) IMU-based parameters, (2) gaze behavior and visual strategies, (3) plantar pressure and postural control, (4) behavioral adaptations from video analysis, and (5) multimodal integration with streamlined clinical protocol. This phased approach allows thorough description of modality-specific analytical procedures while building toward integrated multimodal analysis.

*Rationale for phased publications*: The separate papers serve to thoroughly describe modality-specific signal processing, parameter extraction, and validation procedures—each of which requires substantial methodological detail inappropriate for a single comprehensive paper. Each wearable device has generated an exceptionally large volume of data with fundamentally different characteristics requiring distinct analytical approaches. IMU data (9 locations × 3-axis acceleration and angular velocity × 128 Hz sampling × 15 tasks × 60 participants) require completely different signal processing pipelines, filtering strategies, and parameter extraction methods than eye-tracking data (gaze coordinates, pupil diameter, saccade detection algorithms, fixation identification) or pressure insole data (16 sensors per foot × force-time curves × contact phase segmentation). The analytical requirements, validation procedures, quality control metrics, and interpretation frameworks are unique to each modality and demand specialized expertise. This approach avoids redundancy and allows focused, rigorous reporting of each modality’s unique analytical requirements while doing justice to the richness and complexity of each data type. Additionally, this strategy enables earlier dissemination of clinically useful single-modality findings while the more complex multimodal integration analyses are ongoing.

Importantly, the separate papers are not isolated analyses that ignore the multimodal richness; rather, they provide the methodological foundation and validated parameters necessary for principled multimodal integration in Paper 5. Following the comprehensive analysis of this extensive protocol version, the streamlined clinical version presented in Paper 5 will be optimized for routine clinical implementation. However, presenting this first comprehensive version is essential as it represents a major methodological milestone and provides the empirical foundation for informed, data-driven selection of the minimal effective assessment battery.

All analyses will use open-source software with fully documented pipelines. De-identified processed data and analysis code will be made publicly available upon publication to facilitate reproducibility.

### Results

#### Demographic and clinical characteristics

Demographic and clinical characteristics of the study population are presented in Table 1. We successfully recruited 60 participants (20 per group) with the following characteristics:

*Age and anthropometric matching*: The three groups were well-matched for demographic variables, confirming adequate group comparability. Mean age was similar across groups (HS: 57.9 ± 5.3 years, UV: 59.5 ± 5.5 years, BV: 60.7 ± 11.5 years, *p* = 0.557), with age ranges of 45–65 years (HS), 46–66 years (UV), and 44–83 years (BV). Height did not differ significantly between groups (HS: 172.1 ± 8.4 cm, UV: 173.4 ± 10 cm, BV: 168.3 ± 8.6 cm, *p* = 0.192), nor did body mass index (HS: 24.3 ± 4.1 kg/m^2^, UV: 24.7 ± 3.4 kg/m^2^, BV: 25.3 ± 3.5 kg/m^2^, *p* = 0.711).
*Sex distribution*: The sample included 32 females (HS: 10, UV: 10, BV: 12) and 28 males (HS: 10, UV: 10, BV: 8), with balanced sex distribution across groups.
*Etiology*: For UV patients, the most common etiologies were schwannoma (*n* = 9, 45%), idiopathic (*n* = 5, 25%), traumatic (*n* = 2, 10%), Meniere’s disease (*n* = 2, 10%), zona (*n* = 1, 5%), and cyst (*n* = 1, 5%). For BV patients, the predominant etiology was idiopathic (*n* = 14, 70%), followed by ototoxicity (*n* = 3, 15%), schwannoma (*n* = 1, 5%), Meniere’s disease (*n* = 1, 5%), and Cogan syndrome (*n* = 1, 5%).
*Vestibular-related handicap (DHI)*: DHI scores differed significantly between patient groups, with BV patients reporting higher scores (48 ± 19) than UV patients (28 ± 17) (*p* < 0.001). According to established DHI severity classifications, the BV group’s mean score of 48 corresponds to moderate-to-severe handicap, while the UV group’s mean score of 28 indicates mild-to-moderate handicap. HS participants were not assessed with DHI, as this instrument is designed specifically for individuals with vestibular symptoms.
*Health-related quality of life (SF-36)*: SF-36 scores revealed distinct patterns of physical versus mental health impact across groups.

- *Physical Component Score (PCS):* Significant differences were observed across groups (BV: 39.8 ± 12.2, UV: 50.1 ± 9.2, HS: 52.7 ± 9.6, overall p < 0.001). Post-hoc pairwise comparisons revealed that BV patients had significantly lower PCS than both HS (p < 0.001) and UV patients (*p* = 0.037). UV patients’ PCS did not differ significantly from HS (*p* = 0.537). The BV group’s mean PCS of 39.8 falls approximately one standard deviation below the Swiss population norm (T-score of 50).
- *Mental Component Score (MCS):* Differences across groups were not statistically significant (BV: 43.2 ± 11.2, UV: 40.1 ± 15.4, HS: 48.5 ± 15.4, *p* = 0.134). No significant pairwise differences were detected between any groups.

#### Task completion and functional limitations

Task completion rates across the three groups are presented in Table 3. The vast majority of participants completed all 15 tasks successfully. However, some participants, particularly those with BV, declined certain tasks due to symptom severity and safety concerns. As described in the Methods section, all task declination decisions were patient-initiated based on participants’ own assessment of their ability and safety; the research team never prevented or discouraged task performance, though participants were always explicitly informed of their right to decline any task without consequence.

Tasks most frequently declined were those requiring prolonged single-leg balance, reduced base of support, or dynamic movements perceived as high fall risk. Specifically, Task 12-Wood Beam was declined by 5/20 BV patients (25%) due to fear of falling and the narrow walking surface. Task 2-Pants was declined by 4/20 BV patients (20%) due to inability to maintain single-leg balance while dressing. Task 3-Shoes was declined by 2/20 BV patients (10%) due to difficulty bending and balancing simultaneously. Task 9-Stepladder was declined by 1/20 BV patients (5%) due to fear of falling on the narrow steps.

Notably, no HS participants nor UV ones declined any tasks, indicating that task non-completion was specific to the more severely affected BV population. These task non-completion patterns provide clinically valuable information about the functional limitations imposed by severe bilateral vestibular loss. Task completion data will be analyzed in relation to clinical measures (vHIT gains, caloric responses), patient-reported outcomes (DHI, SF-36), and disease characteristics in subsequent studies to identify predictors of functional limitation and inform personalized rehabilitation strategies.

#### Technical data availability

Technical data availability across sensor modalities is presented in Table 4. Overall, sensor data collection was highly successful, with technical issues affecting a small minority of participants.

*IMU data*: IMU data were unavailable for one BV participant across all tasks due to persistent calibration failure despite multiple troubleshooting attempts. This resulted in *n* = 19 for all IMU-based measures in the BV group. All other IMU sensors (9 sensors × 59 participants = 531 sensor-participant combinations) functioned properly throughout all sessions.

*Eye-tracking data*: Eye-tracking data availability was affected by two distinct technical issues:

*Session-level technical issues (affecting all tasks):* 4 participants could not use eye-tracking for any tasks due to equipment or compatibility issues. Two UV participants experienced technical equipment failures (malfunction of eye cameras or calibration system), and four participants (1 HS, 2 UV, 1 BV) required prescription glasses that were incompatible with the eye-tracking device throughout the session. These participants performed all tasks successfully but without eye-tracking data. This resulted in baseline eye-tracking sample sizes of: HS *n* = 19/20, UV *n* = 16/20, BV *n* = 19/20.

*Task 14-specific visual acuity requirements:* Among participants with otherwise functioning eye-tracking equipment, Task 14-Picture Recognition posed a unique challenge. This task required participants to identify and describe detailed images displayed on windows at several meters, demanding high visual acuity. An additional 8 HS (out of 19 with functional equipment), 4 UV (out of 16), and 3 BV (out of 19) elected to use their prescription glasses specifically for this task to see the images clearly, temporarily precluding eye-tracking. These same participants had successfully completed all other tasks with eye-tracking intact. This reduced Task 14 eye-tracking sample sizes to: HS *n* = 11/19, UV *n* = 12/16, BV *n* = 16/19.

*Protocol design constraints:* Eye-tracking was impossible by design for Task 15-Walk in the dark, which required all participants to wear obscuring welding goggles (marked N/A in Table 4 for all groups). Additionally, Task 13-Uphill/Down included an eyes-closed descent phase, during which eye-tracking data could not be meaningfully collected, though data from the eyes-open ascent phase remained valid.

*Pressure insole data*: Pressure insole data were successfully collected for all participants across all completed tasks with no technical failures. All 60 pressure insole units (20 per group) functioned properly throughout all testing sessions, providing complete bilateral plantar pressure recordings.

## Discussion

This study introduces a new protocol designed to functionally assess individuals with vestibular loss using wearable sensors and ecologically valid tasks that simulate daily life settings. In contrast to traditional laboratory-based approaches focused on reflex testing or constrained gait assessments, our design captures multimodal whole-body and gaze-related behaviors in context-rich environments. Below, we discuss the rationale, key features, implementation feasibility, clinical relevance, and limitations of this approach.

### Participant characterization and group matching

The three study groups were successfully matched for key demographic variables (age: *p* = 0.557; height: *p* = 0.192; BMI: *p* = 0.711), ensuring that observed differences in functional performance could be attributed to vestibular status rather than confounding demographic factors. This successful matching was critical given the protocol’s aim to capture vestibular-specific functional limitations.

As anticipated based on the known pathophysiology of vestibular disorders, clinical outcome measures demonstrated a clear gradient of impairment severity: BV > UV ≥ HS. DHI scores revealed significantly greater vestibular-related handicap in BV patients (48 ± 19) compared to UV patients (28 ± 17, *p* < 0.001), reflecting the more severe functional impact of bilateral vestibular loss. With BV scores in the moderate-to-severe range and UV scores in the mild-to-moderate range, these findings validate the clinical distinction between unilateral and bilateral vestibular loss at the level of patient-reported outcomes, confirming that bilateral deficits impose substantially greater functional burden than unilateral deficits.

SF-36 Physical Component Scores showed the expected pattern of impairment (BV: 39.8 ± 12.2 < UV: 50.1 ± 9.2 ≈ HS: 52.7 ± 9.6, overall p < 0.001), with BV patients falling approximately one standard deviation below population norms—representing clinically meaningful impairment in perceived physical health. Notably, UV patients were statistically indistinguishable from healthy controls (*p* = 0.537), suggesting that unilateral vestibular deficits have limited impact on perceived physical health, likely due to effective central compensation mechanisms. In contrast, mental health scores (MCS) showed non-significant differences (*p* = 0.134), suggesting that while vestibular loss substantially impacts physical functioning and perceived physical health, its effect on mental health and psychological well-being is more variable and less pronounced at the group level, though individual patients may experience significant psychological burden.

Importantly, the preserved physical health-related quality of life in UV patients (PCS comparable to HS) contrasts sharply with their significantly elevated DHI scores, highlighting the DHI’s superior sensitivity to vestibular-specific limitations that may not be captured by generic health status instruments. This discordance underscores the value of condition-specific outcome measures and provides important context for interpreting the functional task performance data collected with our multimodal sensor protocol, which aimed to bridge the gap between generic questionnaires, vestibular-specific self-report measures, and objective functional capacity in ecologically valid settings.

### Conceptual framework and rationale

The development of this protocol was grounded in the long-standing observation that laboratory-based tests do not fully capture the functional impairments experienced by patients with vestibular loss in their daily life. Current clinical tests, while precise, often measure isolated reflexes or basic gait patterns in artificial conditions. In addition to these reflex-based assessments, functional tests for gait and posture do exist and are routinely used in clinical practice. However, these evaluations are also conducted in highly standardized and controlled laboratory environments, which lack the complexity, unpredictability, and multisensory demands of real-world settings. As a result, these paradigms fail to replicate the multisensory integration and context-specific adaptations required for daily-life mobility. To bridge this gap, we initially selected the 10 tasks proposed by Mijovic et al. (29) as a foundation and subsequently expanded and refined the task set based on direct input from patients. This iterative, participatory approach resulted in a total of 15 tasks representing various real-life challenges—such as navigating stairs, carrying load, dual-tasking, walking on uneven terrain, or moving in low-visibility conditions—guided by validated questionnaires (DHI, VADL) and patient experience. This patient-centered design ensured both the ecological validity and clinical relevance of the selected tasks.

### Technical and practical implementation

The multimodal sensor setup included nine IMUs, wearable eye-tracking glasses, and plantar pressure insoles, configured to be worn in a semi-standardized but realistic environment. The IMUs were chosen to provide critical data on body segment kinematics and postural sway, offering a low-cost, valid alternative to force plates as well as complex high-tech camera-based systems (e.g., Vicon Motion Capture System) for balance assessment (20, 34, 36, 37). IMUs have also been successfully applied in vestibular research: Jabri et al. (38) demonstrated that wearable IMUs, combined with machine learning, could automatically classify gait patterns of individuals with vestibular deficits with high accuracy (AUROC up to 0.88), highlighting their sensitivity to subtle differences in arm swing and gait dynamics. Furthermore, portable multi-sensor systems using IMUs have enabled characterization of vestibular-evoked balance responses during real-world locomotion; Foulger et al. (39) used synchronized IMUs on the head, lower back, and ankles, along with stochastic electrical vestibular stimulation (EVS), to reveal phase-dependent modulations of vestibular control during walking outside the laboratory.

In parallel, wearable eye-tracking enables the capture of gaze behavior during ambulation and functional tasks—moving beyond the static, head-restrained paradigms that have long dominated vestibular research. Hooge et al. (40) demonstrated that modern wearable eye trackers can reliably record gaze during dynamic movements such as walking, skipping, and jumping, with most angular errors remaining below 3°, and even during rapid movement tasks, errors rarely exceeded 5.8°. Similarly, Kothari et al. (41) introduced the “*Gaze-in-the-Wild*” dataset, which combines head-mounted eye tracking with inertial sensors to explore eye–head coordination during everyday tasks performed in freely moving individuals, thereby underscoring the potential of wearable systems for capturing ecologically valid visual behavior. Building on this, Bevilacqua et al. (33) developed and validated a wireless magnetic eye-tracking system capable of simultaneously measuring eye and head orientation *in vivo*, with potential applications for assessing VOR function outside traditional lab settings.

In addition, wearable head sensors have been integrated into mobile applications for vestibular rehabilitation. Meldrum et al. (51) reported that a system combining a head-mounted inertial sensor, a smartphone interface, and clinician support software was well accepted by patients with vestibular symptoms, allowing real-time feedback during gaze stabilization exercises. These findings support the growing feasibility of ambulatory vestibular assessment and intervention. Taken together, this multimodal configuration enhances ecological validity by simultaneously capturing gaze, motion, and plantar pressure—enabling integrative analyses of vestibular function during mobility in naturalistic conditions.

Task execution was monitored by a dual-operator team to ensure safety, data integrity, and standardized administration across participants. While technically demanding, the full protocol was completed within approximately 1 h per participant, demonstrating feasibility for implementation in specialized clinical or research settings. Moreover, the results of the analyses will guide the refinement of task selection and sensor use for future applications.

### Strengths and potential applications

A fundamental strength of this protocol lies in its multimodal design conducted in ecologically valid settings, enabling integrated analysis of vestibular function across complementary measurement domains during daily-life activities. This approach directly addresses a key limitation of existing vestibular assessments, which typically measure isolated reflexes or employ single sensor modalities in artificial laboratory conditions, potentially missing important patterns of sensorimotor adaptation that emerge only when considering coordinated responses across multiple measurement domains. Our protocol’s design mirrors the integrative nature of balance control itself. While single-modality assessments provide valuable information, balance emerges from multisensory integration rather than any single sensory channel. Each wearable device in our protocol captures distinct, complementary aspects of the sensorimotor response: IMUs quantify whole-body kinematics and movement smoothness across nine body segments, eye-tracking reveals gaze stabilization strategies and visual compensation mechanisms, pressure insoles characterize weight distribution and postural control dynamics, and video analysis documents observable compensatory behaviors and instability events. By simultaneously recording these diverse data streams during functional tasks simulating daily activities, the protocol enables extraction of objective, interpretable parameters across various domains of real-world mobility.

Multimodal integration therefore represents a primary goal of this protocol. Following single-modality characterization establishing robust parameters within each measurement domain, planned integrated analyses will examine how parameters from different sensor modalities jointly characterize functional impairments, predict clinical outcomes (DHI scores, SF-36, task completion), and identify distinct functional phenotypes through clustering approaches. This multimodal integration will demonstrate the added value of simultaneous multi-sensor assessment beyond any single modality alone, revealing synergistic relationships between gaze behavior, postural control, and whole-body movement that cannot be captured by isolated measurements.

The protocol’s capacity for multimodal data collection in ecologically valid settings offers several important applications. It enables rigorous between-group comparisons (BV vs. UV vs. HS) that account for the multifaceted nature of functional impairment and could be applied longitudinally to track disease progression or rehabilitation effects across multiple outcome dimensions simultaneously.

Another important methodological strength is the independent dual-rating of video recordings by two expert observers (one blinded to participant characteristics) using a scoring grid adapted from validated clinical instruments. This approach will enable assessment of inter-rater reliability for observable functional impairments, complementing objective sensor data with expert clinical observation.

The ecological validity of the protocol also makes it a promising candidate for evaluating interventions such as vestibular implants (11, 52–54), physical therapy (55), sensory substitution devices (56), behavioral strategies (57) or upcoming gene therapy (58) aimed at improving balance and quality of life. Unlike traditional laboratory tests that may not translate to real-world benefit, this protocol’s semi-naturalistic design better captures intervention effects on functional capacity in contexts resembling patients’ lived experiences.

### Limitations and considerations

Despite its strengths, the protocol has several limitations. First, although performed in a realistic setting, it remains semi-structured and supervised and may not fully reflect unsupervised home or community-based activities. Indeed, a fundamental tension in our protocol design lies between standardization (necessary for reproducibility and group comparisons) and true ecological validity (capturing real-world behavior). While our protocol is more naturalistic than traditional laboratory assessments, it still constrains compensatory strategies in ways that reduce ecological authenticity. Specifically, task instructions for some activities explicitly prevented common compensatory behaviors that patients would naturally employ in daily life. For example, Task 2-Pants required standing without external support, sitting, or kneeling—precisely the strategies many BV patients report using at home to dress safely. Similarly, Task 7-Stairs requested not holding handrails when possible, and Task 12-Wood Beam required walking on a narrow surface that patients would typically avoid entirely through environmental route selection. This standardization was methodologically necessary to create reproducible, challenging tasks capable of discriminating between groups. However, it paradoxically reduces ecological validity: by preventing habitual compensation strategies, we may overestimate functional limitation in the constrained test environment while simultaneously failing to capture the most prevalent real-world compensation—avoidance behavior. Importantly, we did not systematically document avoidance strategies or alternative methods patients would use in daily life. Several participants spontaneously mentioned that they would “never do this at home” or would “always sit down” for certain tasks, but these insights were not routinely elicited or recorded. Systematically asking “How would you do this at home?” or “What would you do differently in real life?” would have provided valuable complementary data about real-world functional strategies and activity limitations. This limitation highlights that while our protocol represents an important step toward ecological assessment, truly capturing real-world function would require ambulatory monitoring in patients’ natural environments where they employ their full repertoire of compensation and avoidance strategies without constraint. Future protocol iterations should systematically document both observed compensatory strategies and reported avoidance behaviors, and ultimately progress toward fully ambulatory assessment in patients’ homes and communities using the same sensor setup.

Second, the technical setup—especially the need for wearable calibration, sensor alignment, and synchronized data recording—requires trained personnel and may be challenging to scale in general outpatient settings. Additionally, while each sensor system functioned reliably, the different devices used (IMUs, eye-tracking glasses, and pressure insoles) were not synchronized with each other, which limits the ability to perform precise multimodal temporal alignment across data streams. Furthermore, plantar pressure data were recorded as a single continuous file for the entire session, rather than segmented by task, making task-specific analysis complex and time-consuming in the absence of precise timestamping. While the wearable sensors used in this protocol (IMUs, eye-tracking, pressure insoles) have been validated in various populations, validation specifically in patients with bilateral and unilateral vestibulopathy performing ecologically valid daily-life tasks is limited. The forthcoming sensor analysis papers will establish the discriminative validity and reliability of these measurements in vestibular populations by examining their ability to differentiate between groups and correlate with established clinical measures (DHI, SF-36, vestibular function tests). Another source of variability came from environmental factors: although the setting was semi-standardized, the data collection spanned several months during which weather conditions varied considerably. Because part of the protocol took place outdoors, fluctuations in temperature, lighting, and surface conditions (e.g., dry vs. wet pavement) may have influenced performance and sensor signals, introducing a potential confound (59).

Additionally, although the protocol includes a variety of dynamic and functional motor tasks, it lacks static yet cognitively or sensorimotorically demanding activities that are known to be particularly difficult for individuals with vestibular loss. One notable example is reading while standing or sitting upright, which many patients report as challenging due to oscillopsia or concentration deficits—yet this was not captured in our task battery (60).

Moreover, all tasks were performed in a quiet and uncrowded environment, where participants were alone with the examiner. However, many individuals with vestibulopathies report that their symptoms are significantly exacerbated in noisy, crowded, or visually busy settings—such as supermarkets, public transportation, or social gatherings (61–63). For example, the “normal walking” task, described as easy by all BV participants in our protocol, is often reported as highly effortful or destabilizing when performed in real-world scenarios involving moving crowds or unpredictable environmental stimuli. These ecological stressors are not accounted for in our current design. Fatigue levels were also not accounted for. Indeed, we did not systematically measure fatigue levels before and after protocol completion, which would have provided valuable information about the physical and cognitive demands of the assessment battery.

Our sample included a relatively wide age range, particularly in the BV group (44–83 years), and age-related factors such as sarcopenia, reduced visual acuity, slower processing speed, and general sensorimotor decline may compound vestibular deficits and independently affect task performance. Due to sample size limitations (*n* = 20 per group), we did not perform age-stratified analyses to separate age-related effects from vestibular-specific effects. Future studies with larger cohorts should examine age-specific performance patterns and include age as a covariate in analyses to better characterize the independent contribution of vestibular loss versus age-related sensorimotor decline. Nevertheless, our sample’s age distribution (mean age ~59 years across groups) reflects the typical demographic of vestibular patients seen in clinical practice, which enhances the real-world applicability and generalizability of the protocol to the target patient population.

Another limitation to acknowledge regarding potential confound is the lack of systematic assessment of musculoskeletal pain, particularly arthritic joint pain, which could confound functional mobility measures. While participants were informally asked about pain and discomfort at the session outset, and while we excluded individuals with recent (< 1 year) joint replacement surgery, we did not use standardized pain questionnaires or systematically document arthritis diagnoses. Arthritic pain could lead to movement compensations (e.g., antalgic gait, reduced range of motion) that might be misattributed to vestibular loss in sensor-derived parameters, particularly for pressure insole and IMU data sensitive to gait asymmetries. Future protocol implementations should incorporate validated pain assessment tools (e.g., visual analog scales, Brief Pain Inventory) and document musculoskeletal conditions systematically, with pain considered as a covariate in analyses or used to define exclusion thresholds.

Several limitations regarding participant experience should also be acknowledged. While informal feedback from pilot testing indicated that tasks were perceived as representative of daily challenges and the protocol duration was acceptable, we did not systematically collect structured acceptability data from all 60 participants using standardized questionnaires. This represents a limitation for assessing protocol tolerability. Informal feedback from some participants suggested they would have found the protocol more challenging if performed later in the day, highlighting the potential influence of circadian rhythms and accumulated daily fatigue on vestibular compensation. However, due to scheduling constraints (availability of testing facility, research team, and participants), sessions were conducted throughout the day (08:00–18:00), which may have introduced performance variability. Future iterations of this protocol should include standardized acceptability questionnaires and, ideally, control for time-of-day effects to better assess participant experience and optimize testing conditions.

Finally, psychosocial and emotional dimensions of the disorder—particularly those related to the invisible nature of vestibular disability—are insufficiently reflected by the objective protocol. Many patients describe difficulties with social interactions, feelings of being misunderstood, or withdrawal from previously enjoyed activities (4, 5, 64, 65). While the DHI and, to a lesser extent, the SF-36 capture some of these lived experiences, they are not translated into specific tasks or observable behaviors in the current test battery. Future iterations of the protocol could benefit from incorporating tasks or conditions that elicit these challenges more explicitly, or from integrating real-time patient-reported outcomes during task performance.

### Future directions

This first comprehensive version of the protocol presented here will serve as a foundation for the subsequent development of a streamlined, “turnkey” version, specifically designed for implementation in outpatient clinics and physiotherapy practices.

Future work will aim to optimize the protocol to facilitate broader clinical adoption through several complementary approaches.

*Protocol refinement and clinical implementation*: The final optimized version will retain only the most discriminative tasks and parameters identified through the planned multimodal analyses, resulting in a shorter, more efficient assessment battery while preserving diagnostic sensitivity. Particular attention will be paid to ensuring that physical materials required for task execution (e.g., furniture, obstacles, visual targets) are low-cost, widely available, and easily adaptable to standard clinical environments—without compromising ecological relevance or diagnostic power. Simplification of sensor configuration and development of semi-automated analysis pipelines will further reduce technical barriers to implementation. Moreover, the protocol should be validated in larger cohorts, including diverse clinical subtypes and intervention groups, to establish generalizability and refine normative data.

*Ambulatory assessment in natural environments*: A critical next step would be to complement this semi-standardized protocol with fully ambulatory assessment in patients’ natural environments. The selection of tasks presented here remains inherently subjective, reflecting a balance between ecological validity, experimental control, and feasibility. It would have been impossible to cover the full range of daily-life activities encountered by patients, given the diversity and complexity of real-world environments. As a natural progression, future studies should consider remote or ambulatory assessments conducted directly in patients’ living environments. By equipping participants with the same sensor setup (IMUs, eye-tracking glasses, pressure insoles) and following them through their daily routines at home and in the community, we could capture:

- Compensatory strategies employed without constraint
- Avoidance behaviors and environmental route selection
- Real-world task performance with full access to habitual adaptations
- The gap between constrained test performance and naturalistic functional capacity

This approach would provide a more complete picture of functional limitation, distinguishing between what patients *can* do under standardized conditions versus what they *actually* do in daily life—a distinction critical for personalized rehabilitation and realistic goal setting. This transition to truly ecological conditions would capture nuances of daily functioning that may escape observation in standardized settings, while the insights gathered with the current semi-standardized protocol will guide sensor and analysis strategy selection for such at-home studies.

*Clinical applications and therapeutic evaluation*: Ultimately, by correlating functional biomarkers derived from this protocol with patient-reported outcomes and traditional clinical tests, this approach has the potential to improve diagnosis, personalize rehabilitation strategies, and monitor disease progression in patients with vestibular loss. The protocol’s ecological validity combined with objective sensor-based measurement makes it particularly well-suited for assessing the efficacy of emerging therapeutic interventions such as vestibular implants (11, 52–54) or gene therapy (58), where demonstrating real-world functional benefit is critical for clinical translation.

*Clinical applications and therapeutic evaluation*: Ultimately, by correlating these functional biomarkers with patient-reported outcomes and traditional clinical tests, this approach has the potential to improve diagnosis, personalize rehabilitation, monitor progress in patients with vestibular loss. The protocol’s ecological validity combined with objective sensor-based measurement makes it particularly well-suited for assessing the efficacy of emerging therapeutic interventions such as vestibular implants (11, 52–54) or gene therapy (58), where demonstrating real-world functional benefit is critical for clinical translation.

In summary, the presented protocol provides a robust, patient-centered framework to quantify vestibular-related impairments under realistic conditions. It offers a valuable complement to existing assessment tools and paves the way for more ecologically relevant and responsive vestibular care.

## Conclusion

This protocol presents a comprehensive and multimodal approach to objectively assess the impact of vestibular loss in settings comparable to daily life. By combining wearable technologies and semi-standardized environments, the methodology allows for detailed quantification of motor and gaze behaviors in patients with unilateral and bilateral vestibulopathy. The selection of the tasks, based on patient’s needs, ensures clinical relevance. This type of protocol has the potential to assist clinicians and other vestibular care professionals in both the diagnosis and follow-up of patients by providing objective real-life tasks-based functional assessments, therefore, offering a promising tool for clinical assessment complementary to current reflex-based tests.

## Data Availability

The original contributions presented in the study are included in the article/Supplementary material, further inquiries can be directed to the corresponding author.
