# Supplementary material for: Protocol for assessing functional impairments in patients with unilateral and bilateral vestibulopathy: a novel approach to evaluate the impact of vestibular loss in daily life setting
Source: Front Neurol. 2025 Dec 1;16:1704687. doi: 10.3389/fneur.2025.1704687 (PMC12703974; doi:10.3389/fneur.2025.1704687)
Supplement: Supplementary file 1 [file Data_Sheet_1.docx]

***Supplementary Material***

**Protocol for assessing functional impairments in patients with unilateral and bilateral vestibulopathy: a novel approach to assess the impact of vestibular loss of function in Daily Life Setting.**

| **Task** | | **Instruction** |
| --- | --- | --- |
| **1-Bed** | | “Lie flat on your back and then stand up.” |
| **2-Pants** | | “Put on the pants one leg at a time, without kneeling on the ground or leaning on anything, then take off the pants.” |
| **3-Shoes** | | “Put on the shoes without kneeling down or leaning on anything, then take them off.” |
| **4-Sorting** | | “Store dishes on the shelf by size and color.” |
| **5-Heavy load** | | “Take the bucket in one hand, walk to the turning point, switch the bucket to the other hand, and return. You can switch hands on the fly or by putting the bucket down on the ground.” |
| **6-Bus** | | “Ask for the doors to be opened, get on the bus, sit wherever you like, ask for the stop, then get off.” |
| **7-Stairs up** | **down** | “Walk up the stairs and then descend without holding onto the railing if possible.” |
| **8-Uneven grounds** | | “Walk to the end of this paved road, trying to walk as straight as possible.” |
| **9- Stepladder** | | “Climb 5 steps of this stepladder and then descend.” |
| **10-Tray** | | “Walk with this tray to the finish line, trying not to spill any water.” |
| **11-Walk** | | “Walk to the finish line, at a pace that suits you best and as straight as possible.” |
| **12-Wood Beam** | | “Walk to the end of this beam, turn around on the beam, and come back.” |
| **13-Uphill** | **Down** | “Climb up this inclined plane, if possible, without holding onto the edge. Once you reach the top, turn around and begin the descent with your eyes closed. Halfway done I will tell you when to open them again.” |
| **14-Picture recognition** | | “The three images you drew at random are displayed in the windows. You have one round trip to look at them. At the end of the round trip, tell me what you saw in as much detail as possible. You can slow down but not stop in front of the images.” |
| **15-Walk in the dark** | | “With these dark glasses, walk as straight as possible to the finish line.” |

***Supplementary Table 1****. For each task, the instruction provided to the participant before is detailed.*

| **Task** | **Description** | **Challenge** | **Reason for being discarded** |
| --- | --- | --- | --- |
| **Sink** | The subject goes to the bathroom’s sink, clean their hands 3 times with the eyes closed | - Lack of vision | Negative feedback from patients during pilot study (too easy, not relevant) |
| **Coffee** | The subject buys a drink of their choice at the hospital cafeteria and goes sit at a table | - Navigation through a crowd - Dual task | Involves filming patients and hospital staff who are not participating in the study. |
| **Escalator (down)** | The subject takes the escalator, preferably without holding onto the handrail. They close their eyes halfway and then open them again. | - Lack of vision - Moving environment | Involves filming patients and hospital staff who are not participating in the study. |
| **Revolving door** | The subject goes through the revolving door to exit outside. | - Modification of proprioception (tiles-carpet-asphalt) - Moving environment | Involves filming patients and hospital staff who are not participating in the study. |
| **Outside Walk** | The subject walks on the hospital esplanade. | - Modification of proprioception - Modification of luminosity | Too dependent on weather conditions  Involves filming patients and hospital staff who are not participating in the study. |
| **Sidewalk** | The subject crosses two sidewalks and then returns. | - Shift in center of gravity - Obstacle clearing | Too dependent on weather conditions  Involves filming patients and hospital staff who are not participating in the study. |
| **Foam** | The subject walks on a foam mattress, turns around, and returns. | - Modification of proprioception | Poor ecological validity  Redundant with the “uneven ground” task |
| **Obstacle** | The subject walks 10 meters and must slalom between cones. | - Frequent changes in trajectory | Poor ecological validity |
| **Shopping cart** | The subject fills a shopping cart with groceries and pushes it 6 meters. | - Reduced visual field - Shift in center of gravity | Redundant with “heavy load” task |
| **Walk on the grass** | The subject walks barefoot for 6 meters on grass. | - Modification of proprioception | Too dependent on weather conditions  Involves filming patients and hospital staff who are not participating in the study. |
| **Car** | The subject gets into the car, sits down, fastens his seatbelt, unfastens it, and then gets out again. | - Shift in center of gravity | Redundant with the bus task |

***Supplementary Table 2****.* ***Tasks considered but excluded during iterative protocol development. This table documents the tasks that were initially considered (some adapted from Mijovic et al. (29), others proposed by our team or suggested during patient interviews) but were ultimately excluded from the final protocol. For each task, we provide its description, the intended sensorimotor challenge, and the rationale for exclusion (logistical constraints, ethical considerations, participant feedback, redundancy with other tasks, or poor ecological validity). This transparency regarding the selection process demonstrates that the final 15-task set resulted from systematic evaluation rather than arbitrary selection****.*

Although the initial selection of tasks was considered relevant for assessing functional performance in patients, several of them (which are presented in Supplementary Table 2) had to be discarded during the study design phase. This decision was made for multiple reasons, including logistical constraints (e.g., tasks requiring excessive movement across the hospital or equipment that was not readily available), ethical considerations (e.g., the need to film patients or hospital staff not participating in the study), participant comfort (e.g., stress related to being in crowded areas or under the observation of others), participant feedback (e.g., tasks deemed too easy, not representative of daily challenges, or generating excessive stress), redundancy (e.g., tasks overlapping substantially with other included tasks); and protocol duration (to ensure completion within approximately one hour without excessive fatigue). This documented refinement process demonstrates that our final task set represents a purposeful, evidence-based selection tailored to capture the full spectrum of functional challenges in both UV and BV populations within a feasible testing framework.
